# PemToxTrial: An Observational Study of PEMetrexed-Associated Severe HematoTOXicity in Lung Adenocarcinoma with Bone Metastases

**DOI:** 10.3390/cancers18203281

**Published:** 2026-10-12

**Authors:** Christian Riedel, Christian Neumann, Raban Heller, Arthur Bauer, Patrick Büchele, Konrad Steinestel, Michael Grunert, Jan Sölter, Niklas Gebauer, Annette Arndt, Matthias Müller, Armin Riecke, Hanno Witte

**Affiliations:** 1 Department of Gastroenterology, Bundeswehrkrankenhaus Ulm, Oberer Eselsberg 40, 89081 Ulm, Germany; 2 Department of Hematology and Oncology, Bundeswehrkrankenhaus Ulm, Oberer Eselsberg 40, 89081 Ulm, Germanyarminriecke@bundeswehr.org (A.R.); 3 Lung Cancer Center Ulm, Bundeswehrkrankenhaus Ulm, Oberer Eselsberg 40, 89081 Ulm, Germany; 4 Department for Trauma Surgery and Orthopaedics, Reconstructive and Septic Surgery, Sportstraumatology, Bundeswehrkrankenhaus Ulm, Oberer Eselsberg 40, 89081 Ulm, Germany; 5 Department of Pneumology, Bundeswehrkrankenhaus Ulm, Oberer Eselsberg 40, 89081 Ulm, Germany; 6 Institute of Pathology and Molecular Pathology, Bundeswehrkrankenhaus Ulm, Oberer Eselsberg 40, 89081 Ulm, Germany; 7 Department of Nuclear Medicine, Bundeswehrkrankenhaus Ulm, Oberer Eselsberg 40, 89081 Ulm, Germany; 8 Department of Nuclear Medicine, University Hospital Ulm, Albert-Einstein-Allee 23, 89081 Ulm, Germany; 9 Department of Thoracic Surgery Bundeswehrkrankenhaus Ulm, Oberer Eselsberg 40, 89081 Ulm, Germany; 10 Department of Hematology and Oncology, University Hospital Schleswig-Holstein (UKSH) Campus Lübeck, Ratzeburger Allee 160, 23566 Lübeck, Germany; 11 Department of Hematology and Oncology, University Ulm, Albert Einstein Allee 23, 89081 Ulm, Germany

**Keywords:** bone metastasis, hematotoxicity, adenocarcinoma of the lung, metabolic tumor volume, NSCLC

## Abstract

Introduction: Pemetrexed, an antifolate, is a standard therapy for lung adenocarcinoma (LUAD). Since folic acid is vital for nucleic acid metabolism, disrupting its pathway affects both tumor cells and regenerating healthy cells. The PemToxTrial examined the impact of pemetrexed on severe hematotoxicity and mortality. Methods: This retrospective analysis included 136 LUAD patients (UICC8 stage IVA/B) receiving palliative treatment. Ninety-two patients received pemetrexed-based therapy, while 44 received pemetrexed-free treatment (68 and 38 with immune-checkpoint inhibitors). Bone metastasis (BM) was present in 47 patients in the pemetrexed group and 22 in the comparison group. The primary endpoint was grade III–V hematotoxicity; secondary endpoints included mortality, progression-free survival (PFS), and overall survival (OS). Hematologic outcomes following a subsequent change in clinical practice were prospectively assessed in a post-implementation cohort of 15 patients with bone-metastatic LUAD receiving pemetrexed-free regimens. Results: Median age was 66 years, 59.6% were male and median follow-up was 13.5 months. Grade III-V hematotoxicity was most frequent (81%) in patients with BM receiving pemetrexed (*n* = 47), significantly higher than in other subgroups (*p* < 0.0001). Mortality was also increased in this group (66% vs. 29%, *p* < 0.0001), with hematotoxicity directly contributing to death in 42% versus 11.5% of cases. PFS was significantly inferior (5.2 vs. 8.6 months). Multivariable analysis identified BM as the only independent predictor of severe hematotoxicity and mortality. Only a mutational burden of ≥4 potential driver alterations was associated with inferior PFS. Bone-derived total lesion glycolysis (TLG) on PET-CT strongly correlated with high-grade hematotoxicity (*p* < 0.0001). Conclusions: The PemToxTrial identified a strong association between pemetrexed exposure, bone metastases, and severe hematotoxicity, supporting further prospective evaluation of bone metastasis status as a potential clinical risk factor for treatment-associated hematotoxicity.

## Simple Summary

Despite years of use, there is currently no evidence regarding the extent to which bone metastases affect the incidence of side effects during pemetrexed therapy. Based on clinical observations, a retrospective study was conducted to investigate the incidence of severe hematopoietic side effects in patients with lung adenocarcinoma in order to identify patients at risk for these events. In the future, these patients might benefit from a treatment regimen without pemetrexed or from closer monitoring during therapy, in line with a personalized treatment approach.

## 1. Introduction

Pemetrexed constitutes an essential therapeutic agent within the established therapeutic regimen for treating adenocarcinoma of the lung (LUAD) [1]. Initial approvals for immunotherapeutic approaches were extended to combinations involving a platinum derivative and pemetrexed, exemplified by the KEYNOTE-189 trial [2]. Recently, pemetrexed has been used more frequently in combination with amivantamab (MARIPOSA-2) in EGFR-altered non-small-cell lung cancer (NSCLC) [3]. Pemetrexed belongs to the class of antifolates, exerting its effects within the spectrum of folate antagonists. Folate, a critical vitamin, plays a pivotal role in the salvage pathway of nucleic acid metabolism. Consequently, perturbations in folate metabolism assume paramount significance for cellular proliferation, affecting not only neoplastic cells but also normal cells characterized by heightened regenerative turnover rates [4]. Integration of these therapeutic paradigms has markedly ameliorated the prognosis for NSCLC within palliative care contexts. However, the implementation of such strategies poses challenges to clinicians, particularly in managing immune checkpoint inhibitor (ICI)-associated toxicities, some of which can prove fatal [5].

Notwithstanding, severe to fatal toxicities attributable to conventional chemotherapy, as delineated by the Common Toxicity Criteria (CTCAE v.6.0), persist in routine clinical settings during induction therapy [6]. Clinically, a notable observation has been the incidence of fatal hematotoxicities, especially in cases of osseous metastasized LUAD treated with pemetrexed. This necessitates transfusions, heightens the risk of severe hemorrhagic events, or precipitates neutropenic sepsis, thereby potentially augmenting patient mortality. Yet, the correlation between these occurrences remains inadequately elucidated in the extant literature [7].

In KEYNOTE-189, hematotoxicity represented the most common severe adverse events (CTCAE grade III–V), including thrombocytopenia (7.9%), neutropenia (15.8%), and anemia (16.3%) [2]. However, no dedicated subgroup analysis of patients with bone metastases was performed, and consequently no treatment restrictions or alternative chemotherapy recommendations exist for this population [1].

The PemToxTrial endeavors to enhance patient safety by exploring the impact of pemetrexed in LUAD patients with bone metastases, focusing on severe hematotoxicity and associated mortality. Alongside clinical parameters, comprehensive molecular analyses and metabolic tumor burden assessments were conducted to identify potential biomarkers conducive to risk stratification for hematotoxicity.

## 2. Methods

### 2.1. Patients

For this analysis, all patients aged ≥18 years with histologically confirmed LUAD treated at the Lung Cancer Center Ulm between 2018 and 2024 were screened (data cut-off 5 November 2024). An interim analysis prompted the Lung Cancer Center Ulm to discontinue recommending pemetrexed-containing regimens for patients with UICC stage IVA/B disease and bone metastases in tumor board discussions [8]. Following an interim analysis of the retrospective cohort, pemetrexed-containing regimens were no longer routinely recommended for patients with bone-metastatic LUAD at our center. Subsequently, 15 consecutive patients with bone-metastatic LUAD were prospectively observed between December 2024 and September 2025 after implementation of this change in clinical practice. Severe hematotoxicity events (CTCAE grade III–V) and hematologic supportive-care requirements were prospectively recorded. This cohort was considered an observational post-implementation cohort and was not designed as an independent validation cohort or as a comparative interventional study. Clinical data were obtained from the electronic medical records of the Bundeswehr Hospital Ulm (Supplementary Table S1). Eligibility criteria included an Eastern Cooperative Oncology Group Performance Status (ECOG-PS) of 0–2 and measurable disease as per RECIST v1.1 criteria [9]. Of 338 initially screened patients, 202 were excluded. Of these, 134 did not receive systemic therapy, including 77 patients undergoing primary surgery without adjuvant systemic therapy and 57 patients receiving radiotherapy or best supportive care. An additional 32 patients received targeted therapy only, 3 received simultaneous treatment for a second malignancy, and 33 were lost to follow-up outside our institution (Supplementary Figure S1). The final retrospective study cohort therefore comprised 136 patients. Patients with brain metastases were included irrespective of local treatment status at therapy initiation. The prospective validation cohort comprised 15 cases.

### 2.2. Study Design and Subgroups

This retrospective, single-center analysis categorized cases into four distinct subgroups. The first subgroup included patients treated with pemetrexed who presented with bone metastases at initial diagnosis (*n* = 47), while the second subgroup consisted of patients treated with pemetrexed without bone metastases (*n* = 45). Timely supplementation with folic acid and vitamin B12 was ensured prior to the initiation of pemetrexed therapy in accordance with the institutional local guidelines. Two additional subgroups included patients who did not receive pemetrexed, one with bone metastases (*n* = 22) and the other without bone metastases (*n* = 22; Supplementary Figure S1). Comprehensive details regarding the clinicopathological evaluation can be found in the Supplementary Materials.

The study was conducted in compliance with the Declaration of Helsinki and the International Conference on Harmonization Good Clinical Practice (ICH-GCP) guidelines. Ethics approval was obtained from the University of Ulm Ethics Committee (reference no. 112-23).

### 2.3. Endpoints

The primary endpoint of this study was the incidence of severe grade III–V hematologic toxicities, assessed according to the Common Terminology Criteria for Adverse Events (CTCAE v.6.0). Secondary endpoints included hematotoxicity-associated mortality, overall mortality, progression-free survival (PFS), and overall survival (OS).

Overall mortality was defined as death from any cause during follow-up. Hematotoxicity-related mortality was defined as death occurring in the context of severe treatment-associated cytopenia, febrile neutropenia/sepsis, or bleeding without an alternative dominant cause. Such events were classified as CTCAE grade V hematotoxicity. Accordingly, grade V hematotoxicity and hematotoxicity-related mortality represented the same endpoint and were not analyzed as separate outcomes.

### 2.4. Assessment of Hematotoxicity

To obtain a comprehensive assessment of hematotoxicities, multiple parameters were systematically recorded throughout the study. Hematologic toxicities were classified according to the CTCAE, with severity graded on a scale from I to V. Particular emphasis was placed on the occurrence of severe hematotoxicity events (grade III–V). Standard blood count parameters, including hemoglobin (for anemia), leukocytes/neutrophils (for neutropenia), and platelets (for thrombocytopenia), were measured at predefined time points before and during (immuno)chemotherapy (Supplementary Figure S2). This approach enabled the temporal evaluation of hemoglobin levels and their trends throughout treatment.

In addition to laboratory values, clinical symptoms such as bleeding and infection events, along with therapeutic interventions including blood transfusions and the administration of growth factors (G-CSF), were documented. These data allowed for a more comprehensive assessment of the clinical relevance of hematotoxicity.

Furthermore, all dose reductions, treatment interruptions, discontinuations, and deaths attributed to hematologic adverse events were recorded. These data provided valuable insights into the impact of hematotoxicity on therapeutic efficacy and overall patient management.

### 2.5. Molecular Profiling

For molecular analyses, formalin-fixed, paraffin-embedded (FFPE)-derived DNA underwent standardized pre-analytical processing including macrodissection when required. Library complexity and fragment size distributions were assessed prior to sequencing to ensure uniform input quality. Next-generation sequencing (NGS) runs were performed on a NextSeq 550Dx sequencer (Illumina, San Diego, CA, USA) and subjected to internal quality metrics, including mean coverage depth, uniformity, and on-target rates, according to TruSight Oncology 500 (TSO500, Illumina, San Diego, CA, USA) specifications. The genes that were included in the TSO500 panel were specified in Supplementary Table S2. Only variants passing internal quality thresholds were considered for downstream analyses. Copy number alterations were reported if exceeding platform-specific confidence scores. Microsatellite instability (MSI) and tumor mutational burden (TMB) estimates were derived from validated internal algorithms within the TSO500 Local App environment. Details on data processing are provided in the Supplementary Materials.

### 2.6. Metabolic Tumor Volume and Radiomics

Metabolic tumor volume (MTV) was measured using positron emission tomography-computed tomography (PET-CT) scans, which were analyzed with the radiomics software LIFEx (version 25.06.1) [10]. MTVs were derived using the LIFEx software with semi-automatic segmentation based on a threshold of >40% of the lesion-specific SUVmax. Segmentations were performed independently by two investigators under the supervision of a board-certified nuclear medicine physician, with inter-observer variability below 1%. MTV and total lesion glycolysis (TLG; MTV*mean SUV) were calculated and integrated into subsequent statistical analyses assessing associations with hematotoxicity, PFS, OS, and mortality. All analyses were conducted in accordance with EANM (European Association of Nuclear Medicine) guidelines. Further details on image processing and validation procedures are provided in the Supplementary Materials.

### 2.7. Statistical Analysis

Statistical analyses were performed using GraphPad Prism 9 (GraphPad Software, San Diego, CA, USA), R version 4.5.2, and SPSS version 26 (IBM, Armonk, NY, USA). Categorical variables were compared using Fisher’s exact test (two-sided), and continuous variables were compared using the Wilcoxon rank-sum test, as appropriate.

The primary endpoint, severe hematotoxicity (CTCAE grade III–V), was analyzed as a binary outcome using logistic regression. Overall mortality was analyzed analogously as a binary outcome. Univariable logistic regression models were initially used to assess associations between individual clinical and treatment-related covariates and the respective outcome. Multivariable logistic regression models were subsequently constructed to evaluate independent associations, with effect estimates reported as odds ratios (ORs) and corresponding 95% confidence intervals (CIs).

To formally assess effect modification by bone metastasis status, the primary logistic regression model for severe hematotoxicity included the main effects of pemetrexed exposure (yes vs. no), bone metastasis status (present vs. absent), and a multiplicative pemetrexed × bone metastasis interaction term. Patients without pemetrexed exposure and without bone metastases constituted the reference category. The interaction term was used to test whether the association between pemetrexed exposure and severe hematotoxicity differed according to bone metastasis status. The adjusted interaction model additionally included age, ECOG performance status, proton-pump inhibitor use, vitamin D supplementation, bisphosphonate therapy and the presence of additional metastatic sites.

Progression-free survival (PFS) and overall survival (OS) were analyzed as time-to-event outcomes. Survival distributions were estimated using the Kaplan–Meier method and compared between groups using the log-rank test. Where multivariable time-to-event analyses were performed, Cox proportional hazards regression was used, with associations reported as hazard ratios (HRs) and corresponding 95% CIs.

In a sensitivity analysis, platinum backbone (cisplatin vs. carboplatin) was additionally included as a covariate to account for differences in platinum use across treatment subgroups.

Missing values were not imputed. Analyses were performed using available cases, with complete-case analysis applied to multivariable models requiring covariates with missing observations. The number of evaluable patients is reported where data were not available for the entire cohort. All statistical tests were two-sided. Secondary and exploratory analyses were considered hypothesis-generating, and no formal adjustment for multiple testing was performed.

## 3. Result

### 3.1. Patient Population and Baseline Characteristics

Between 2018 and 2024, a total of 136 patients with metastatic LUAD were enrolled in this study. Of these, 92 patients (47 with osseous metastasis) received pemetrexed as part of their treatment regimen, while 44 patients were treated with alternative chemotherapy and/or immunotherapy. Baseline demographic and clinical characteristics, including age, sex, ECOG performance status, smoker status, B-symptoms and metastatic sites across the different subgroups are outlined in Supplementary Table S8. Additional information concerning concomitant treatment with proton pump inhibitors (PPI) and vitamin D supplementation is provided in the Supplementary Section (Supplementary Table S3). The median age of patients was 66 years (range, 35–87 years), and 59.6% were male. The majority of patients presented with an ECOG performance status of 1–2 (61.7%) and 46% were active smokers, 36% were former smokers and 18% never previously smoked (Supplementary Figure S3a). Regarding the intensity of nicotine abuse, the median number of pack-years was 25 (range 0–150). The distribution of metastatic sites is provided in Supplementary Figure S3b and Supplementary Table S8. Apart from the subgroup-defining feature of bone metastases, the distribution was otherwise well balanced among the different subgroups.

### 3.2. Treatment Characteristics

The distribution of first-line treatment regimens across the four predefined subgroups (PemOss, PemNoOss, NoPemOss, and NoPemNoOss) is depicted in Supplementary Figure S3c. Among the pemetrexed-treated subgroups (PemOss and PemNoOss), the majority of patients received a cisplatin-based platinum doublet combined with an ICI, most frequently according to the KEYNOTE-189 regimen (68% and 62%, respectively) [2]. Only a smaller proportion of patients within these groups were treated with alternative strategies, including platinum doublets (28% and 31%) with or without bevacizumab, or regimens following the POSEIDON protocol (4% and 7%) [11].

In contrast, treatment in the non-pemetrexed subgroups (NoPemOss and NoPemNoOss) was more heterogeneous, comprising a broader range of first-line regimens with immunotherapeutic backbones, including atezolizumab-based protocols (IMpower 130/150; 45% and 18%) and cemiplimab (EMPOWER-Lung 3), as well as combination approaches such as durvalumab plus tremelimumab (POSEIDON; 18% and 9%) or nivolumab plus ipilimumab (CHECKMATE 9LA; 14% and 27%) [11,12,13,14,15]. This pattern reflects the ongoing shift toward non-pemetrexed-based (immuno-)chemotherapy approaches in advanced LUAD. Cisplatin was more frequently used in selected pemetrexed-treated patients (52%), whereas carboplatin predominated overall (57%). Notably, most patients received ICI-containing regimens (106/136; 78%), underscoring chemo-immunotherapy as the prevailing first-line standard in metastatic LUAD. Details on subsequent treatment lines are provided in the Supplementary Materials (Supplementary Table S4); the median number of treatment lines was 2 (range, 1–5).

### 3.3. Survival Analysis

**Figure 1:**
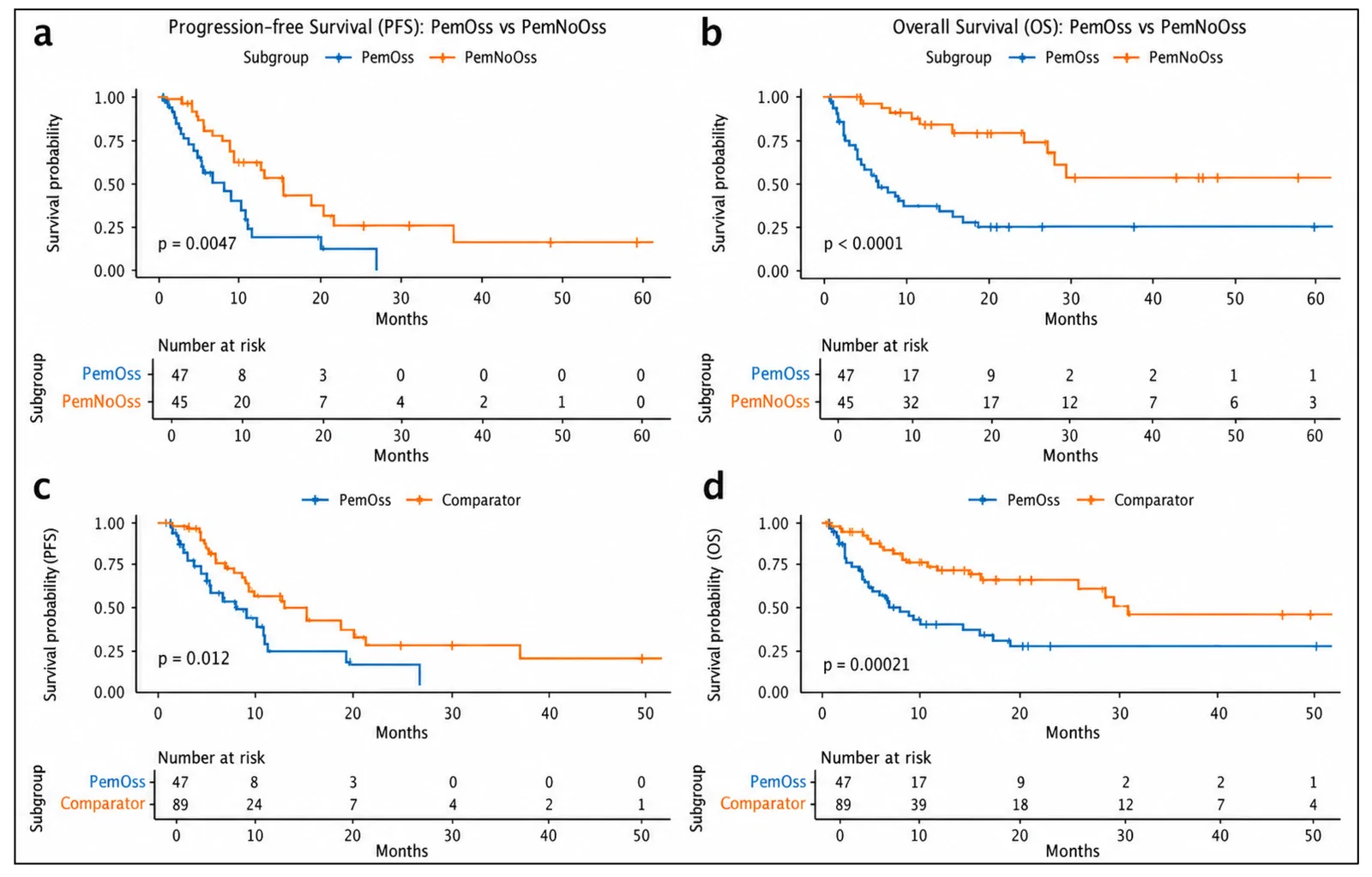
Progression-free and overall survival according to pemetrexed treatment and osseous metastases. (**a**,**b**) Kaplan–Meier curves comparing patients with osseous metastases treated with pemetrexed (PemOss) with patients without osseous metastases treated with pemetrexed (PemNoOss). Patients in the PemOss subgroup showed significantly inferior progression-free survival (PFS; *p* = 0.0047, panel (**a**)) and overall survival (OS; *p* < 0.0001, panel (**b**)). (**c**,**d**) Comparison of the PemOss subgroup with the pooled control cohort (Comparison group containing PemNoOss + NoPemOss + NoPemNoOss). Similarly, PemOss patients demonstrated inferior PFS (*p* = 0.012, panel (**c**)) and OS (*p* = 0.00021, panel (**d**)). Numbers at risk are displayed below each graph.

Kaplan–Meier analyses revealed distinct survival patterns between the predefined subgroups (Figure 1). Median follow-up was 13.5 months across all subgroups. Patients receiving pemetrexed-based therapy in the presence of bone metastases (PemOss) experienced a markedly shorter PFS compared with those without osseous involvement (PemNoOss) (Figure 1a; log-rank *p* = 0.0047; HR = 0.45; 95% CI [0.25–0.82]). This early divergence of the survival curves persisted throughout follow-up. A similar trend was observed for OS, which was significantly reduced in the PemOss subgroup compared with PemNoOss (Figure 1b; log-rank *p* < 0.0001; HR = 0.22; 95% CI [0.11–0.43]).

When all remaining patients (PemNoOss, NoPemOss, and NoPemNoOss) were pooled into a single comparator group, PFS remained significantly shorter in the PemOss cohort (Figure 1c; log-rank *p* = 0.012; HR = 0.51; 95% CI [0.3–0.87]). Likewise, OS was markedly inferior in the PemOss subgroup compared to the combined non-PemOss population (Figure 1d; log-rank *p* = 0.00021; HR = 0.38; 95% CI [0.23–0.65]).

Supplementary analyses further corroborated these findings. In the three-group comparison including PemOss, PemNoOss, and the aggregated non-pemetrexed population (NoPem = NoPemOss + NoPemNoOss), patients in the PemOss subgroup exhibited the poorest outcomes, whereas PemNoOss patients showed the most favorable survival (Supplementary Figure S4a,b; PFS: log-rank *p* = 0.017; HRs = 0.68; 95% CIs [0.33–1.40]; OS: log-rank *p* < 0.0001; HRs = 0.82; 95% CIs [0.44–1.53]).

An expanded analysis across all four subgroups (PemOss, PemNoOss, NoPemOss, and NoPemNoOss) confirmed these results (Supplementary Figure S4c,d). Overall, both PFS and OS differed significantly between the groups (PFS: log-rank *p* = 0.042; HRs = 0.62; 95% CIs [0.25–1.57]; OS: log-rank *p* < 0.0001; HRs = 0.68; 95% CIs [0.28–1.65]), consistently identifying PemOss as the subgroup with the most unfavorable prognosis. Due to the limited number of cases in the non-pemetrexed-treated subgroups, the survival curves separate only incompletely when all four subgroups are displayed together. Response rates towards 1st line treatment are summarized in Supplementary Table S5.

Collectively, these analyses indicate that the combination of pemetrexed-based chemotherapy and osseous metastases was associated with a poorer clinical course in terms of disease progression and overall survival. In contrast, patients receiving pemetrexed in the absence of bone metastases showed more favorable PFS and OS outcomes in this cohort.

### 3.4. Hematologic Toxicity and Bone Metastasis

To explore the association between bone metastases and the occurrence of severe hematotoxicity, several factors influencing bone metabolism were assessed, including vitamin D supplementation, proton pump inhibitor therapy and bisphosphonate treatment.

**Figure 2:**
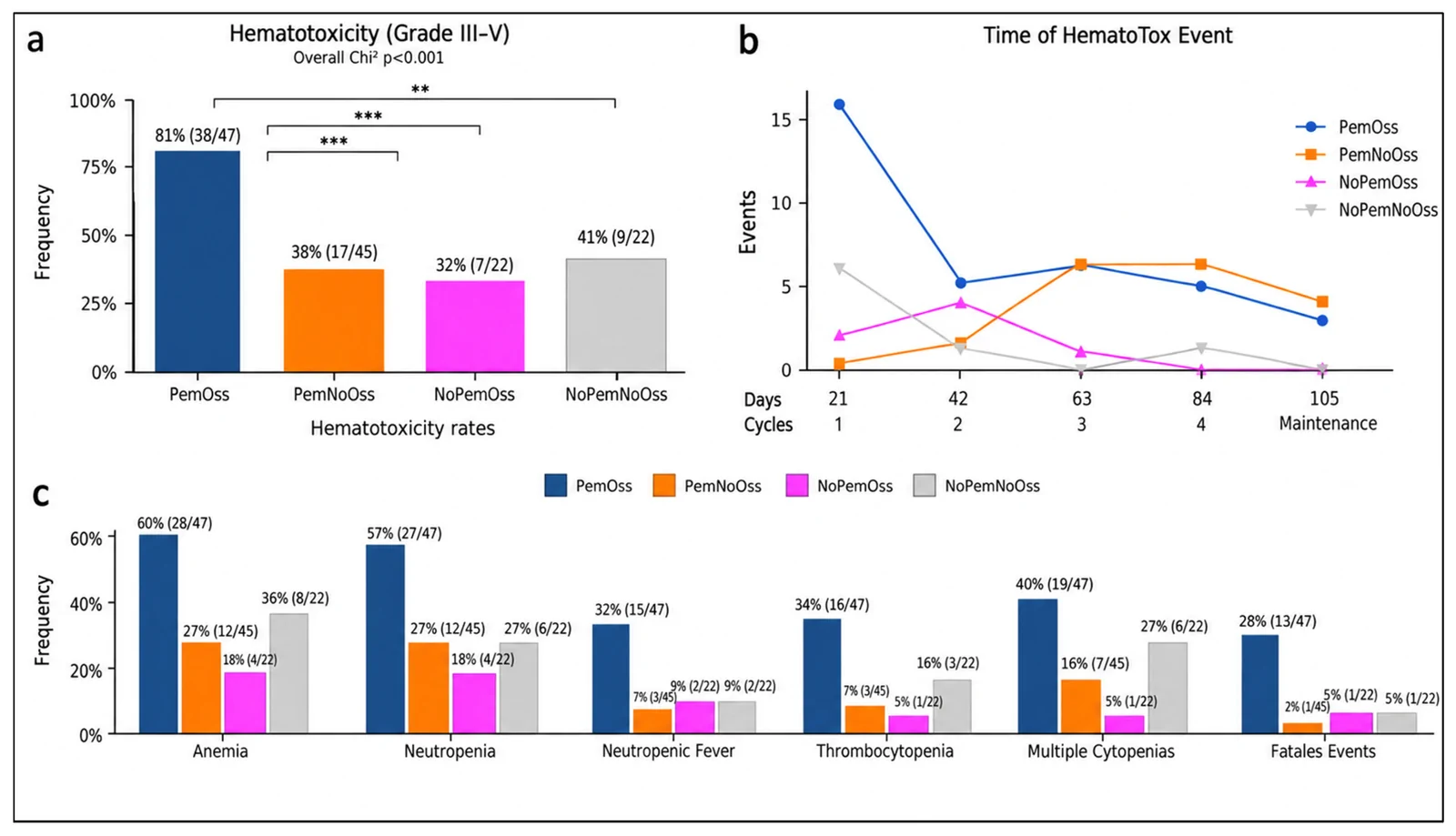
Incidence, temporal dynamics, and spectrum of hematologic toxicity across treatment subgroups. (**a**) Frequency of grade III–V hematotoxicity among the four study subgroups (PemOss, PemNoOss, NoPemOss, NoPemNoOss). Patients receiving pemetrexed with concurrent osseous metastases (PemOss) exhibited the highest rate of severe hematotoxicity (81%, *p* < 0.0001). (**b**) Temporal distribution of hematotoxic events across treatment cycles. In the PemOss subgroup, most events occurred during the initial treatment cycle, whereas in PemNoOss and NoPemOss patients, hematotoxicity was more evenly distributed throughout subsequent cycles. (**c**) Composition of hematologic adverse events by subtype and treatment group, illustrating the relative frequencies of anemia, thrombocytopenia, febrile neutropenia, and multilineage cytopenia. The overall burden of hematologic toxicity was highest in the PemOss subgroup. Statistical significance: ** *p* < 0.01; *** *p* < 0.001. Pairwise comparisons are indicated by brackets.

Severe hematologic toxicity (grade III–V) occurred in 81% (38/47) of pemetrexed-treated patients with bone metastases, compared to 38% (17/45) in those without bone metastases and 32% and 41% in the non-pemetrexed-treated subgroups with and without bone metastases, respectively (Figure 2a and Table 1).

To formally test whether bone metastasis status modified the association between pemetrexed exposure and severe hematotoxicity, a logistic regression model including pemetrexed exposure, bone metastasis status, and a pemetrexed × bone metastasis interaction term was performed. A significant interaction between pemetrexed exposure and bone metastasis status was observed (interaction OR = 11.35, 95% CI [2.39–53.93], *p* = 0.002), indicating that the association between pemetrexed exposure and severe hematotoxicity differed according to bone metastasis status. Importantly, this interaction remained significant after adjustment for age, ECOG performance status, proton pump inhibitor use, vitamin D supplementation, bisphosphonate therapy and the presence of additional metastatic sites (adjusted interaction OR = 6.53, 95% CI [1.13–37.70], *p* = 0.036).

Because cisplatin was more frequently used in the pemetrexed-treated subgroups, platinum backbone was additionally evaluated as a potential treatment-related confounder. Among patients receiving platinum-based therapy, cisplatin versus carboplatin was not associated with severe hematotoxicity in univariable analysis (OR = 1.05, 95% CI 0.53–2.11, *p* = 0.883). Addition of platinum backbone to the interaction model did not materially alter the pemetrexed × bone metastasis interaction (interaction OR = 12.07, 95% CI 2.40–60.59, *p* = 0.0025), while platinum type itself was not significantly associated with severe hematotoxicity (OR = 0.61, 95% CI 0.26–1.43, *p* = 0.254). In a sensitivity analysis additionally adjusting for age, ECOG performance status, proton-pump inhibitor use, vitamin D supplementation, bisphosphonate therapy, and additional metastatic sites, the interaction remained statistically significant (adjusted interaction OR = 6.58, 95% CI 1.01–42.97, *p* = 0.049), whereas platinum backbone remained unrelated to severe hematotoxicity (adjusted OR = 1.18, 95% CI 0.39–3.53, *p* = 0.771; Supplementary Figure S5).

Longitudinal assessment over the treatment course revealed that 50% (19/38) of hematotoxicity events in the pemetrexed + bone metastasis subgroup occurred during the first cycle (within 21 days). Thereafter, event rates approximated those of other subgroups (Figure 2b).

Anemia, neutropenia, and thrombocytopenia are displayed as overlapping lineage-specific toxicity categories and are not mutually exclusive. Individual patients may therefore contribute to more than one category. Multilineage hematotoxicity indicates involvement of two or more hematopoietic lineages during the respective treatment-associated toxicity episode; lineage-specific nadirs were not required to occur on the same day.

The most common hematologic toxicities were anemia (60% vs. 27%) and neutropenia (57% vs. 25%). Furthermore, neutropenic fever (32% vs. 8%), thrombocytopenia (34% vs. 8%), and multiple cytopenias (40% vs. 16%) were more frequent among pemetrexed-treated patients with bone metastases. Grade V hematotoxicity, corresponding to hematotoxicity-related mortality as predefined in the Methods, occurred in 9 of 47 patients (19.1%) in the PemOss subgroup, compared with 1 of 45 (2.2%) in PemNoOss, 1 of 22 (4.5%) in NoPemOss, and 1 of 22 (4.5%) in NoPemNoOss (Figure 2c and Table 1).

The lineage-specific hematotoxicity categories were not mutually exclusive, as individual patients could experience cytopenias affecting multiple hematopoietic lineages during the same treatment-associated hematotoxic episode. Among the 71 patients with severe hematotoxicity, 38 (53.5%) had involvement of a single lineage, 13 (18.3%) of two lineages, and 20 (28.2%) of all three lineages. Accordingly, 33/71 patients (46.5%) experienced multilineage cytopenia. Multilineage involvement was particularly frequent in the PemOss subgroup, occurring in 19/47 patients (40.4%) overall and in 19/38 (50.0%) of those who developed severe hematotoxicity. As lineage-specific nadirs may occur at different time points during chemotherapy, multilineage cytopenia did not require the nadirs of the affected hematopoietic lineages to occur on the same day.

Supportive interventions were also markedly increased in the PemOss subgroup. A total of 51 red blood cell transfusions were administered to 24 patients, and 22 platelet concentrates to 11 patients. Among the remaining 89 patients, only 48 red blood cell and 3 platelet units were transfused in 22 individuals. The need for G-CSF support was roughly twofold higher in the PemOss subgroup (36% vs. 18%). Details of transfusion frequency, G-CSF use, and toxicity grades are summarized in Table 1.

**Figure 3:**
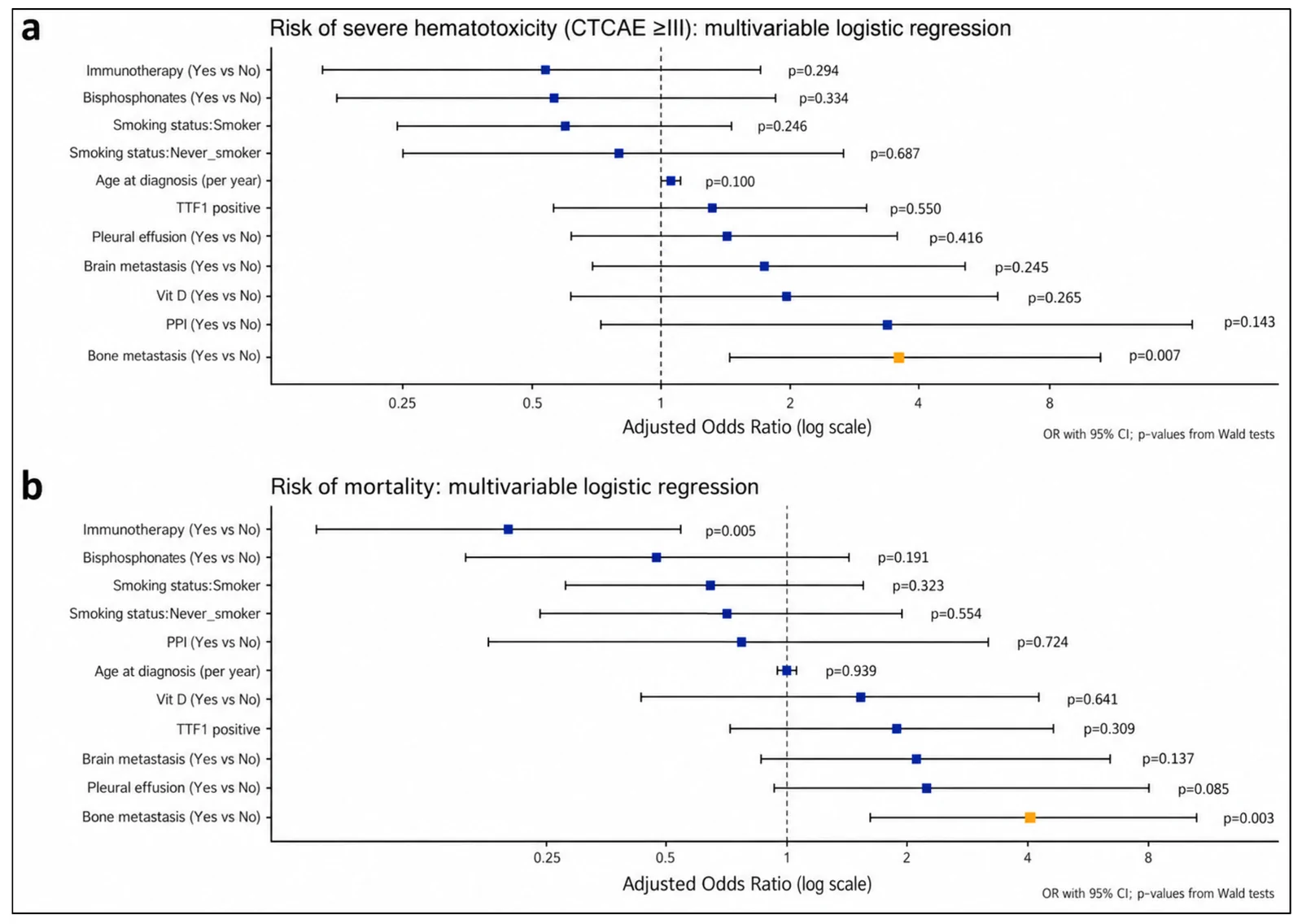
Multivariable logistic regression analyses assessing the risk of severe hematotoxicity and mortality. (**a**) Forest plot showing adjusted odds ratios (OR) with 95% confidence intervals for the association between clinical and treatment-related variables and the risk of severe hematotoxicity (CTCAE grade ≥ III). Bone metastases were independently associated with an increased risk of severe hematotoxicity (*p* = 0.007), whereas none of the other evaluated factors—including immunotherapy, bisphosphonate use, smoking status, or age—showed a statistically significant association. (**b**) Multivariable model evaluating predictors of mortality. Bone metastases were significantly associated with higher mortality risk (*p* = 0.003), while concomitant immunotherapy was linked to improved survival outcomes (*p* = 0.005). All other variables did not reach statistical significance among those experiencing severe hematotoxicity.The vertical dashed line indicates an odds ratio (OR) of 1, representing no association with the outcome. Squares indicate adjusted odds ratios, and horizontal lines represent the corresponding 95% confidence intervals. ORs are displayed on a logarithmic scale.

Upon multivariable analysis adjusting for age, smoking status, TTF1 expression, PPI therapy, vitamin D and bisphosphonate use, as well as brain or pleural metastases, the presence of bone metastases independently predicted severe hematotoxicity (grade III–V) in pemetrexed-treated patients (Figure 3a; adjusted OR = 3.93, 95% CI [1.64–9.4], *p* = 0.007). In a subsequent model, bone metastases also emerged as an independent predictor of mortality (Figure 3b; OR = 5.34, 95% CI [2.12–13.48], *p* = 0.003). As expected, ICI administration significantly reduced mortality (*p* = 0.005), further supporting the robustness of the findings.

### 3.5. Safety

In addition to hematologic toxicity, clinically relevant severe non-hematologic adverse events (grade ≥ 3) occurring during first-line treatment were assessed (Supplementary Table S6). This analysis was restricted to documented severe adverse events and was not intended to capture the complete spectrum of all-grade treatment-related toxicity. Mucositis occurred in 13% of PemOss and 11% of PemNoOss patients. Acute kidney injury was observed in 11% of PemOss and 9% of NoPemOss patients, while pneumonia/pneumonitis occurred in 6% of PemOss and 4% of NoPemNoOss patients. Grade ≥3 colitis/diarrhea occurred in 4% of PemOss, 4% of NoPemOss, and 14% of NoPemNoOss patients. Cutaneous toxicity/allergic reactions were uncommon and occurred in 2% of PemNoOss and 9% of NoPemOss patients. Other severe non-hematologic adverse events occurred only sporadically. Overall, no accumulation of severe non-hematologic adverse events was observed in the PemOss subgroup comparable to the excess of severe hematotoxicity.

### 3.6. Molecular Characteristics of the Cohort

NGS–based molecular profiling was performed in 90 patients to characterize the genomic landscape of the cohort (Supplementary Figure S6a). The most frequent alterations involved *KRAS* (38%, *KRAS* G12C was the predominant variant) and *TP53* (29%), followed by *STK11*, *CDKN2A*, and *KEAP1*, consistent with established LUAD mutational patterns. Subgroup-specific distributions are shown in Supplementary Figure S6a. None of the twelve most frequently mutated genes was significantly associated with severe hematotoxicity, indicating genomic independence of toxicity risk (Supplementary Figure S6b).

Mutation frequencies were comparable between pemetrexed-treated and non-pemetrexed patients and did not differ between cases with or without high-grade hematotoxicity. The number of potential driver alterations per case was evaluated (Supplementary Figure S7); univariate analyses demonstrated an association between ≥4 driver alterations and inferior PFS, but no association with severe hematotoxicity (Supplementary Figure S8a,b).

PD-L1 TPS was available for 108 patients, of whom 36 (33.3%) had high expression (TPS ≥50%). At the individual-patient level, severe hematotoxicity occurred in 18/36 patients (50.0%) with TPS ≥50% and 38/72 patients (52.8%) with TPS <50% (OR = 0.89, *p* = 0.840). Similarly, among pemetrexed-treated patients, severe hematotoxicity occurred in 13/21 (61.9%) patients with TPS ≥50% and 29/47 (61.7%) with TPS <50% (*p* = 1.000). PD-L1-high status was also not significantly associated with PFS (HR = 0.65, 95% CI 0.31–1.34, *p* = 0.238) or OS (HR = 0.73, 95% CI 0.36–1.50, *p* = 0.394; Supplementary Figure S9, Supplementary Table S8).

Overall, the molecular profile of this cohort reflects canonical LUAD biology, with neither specific genomic alterations nor PD-L1 expression predicting pemetrexed-associated severe hematotoxicity. Detailed analyses are provided in the Supplementary Results.

### 3.7. Metabolic Tumor Volume Assessment

Metabolic tumor volume (MTV) was quantified using PET-CT scans and analyzed with the LIFEx radiomics software (Supplementary Figure S10). MTV measurements were available for 108 patients, with a median MTV of 34.01 cm^3^ (range, 1.18–489.34 cm^3^) across the cohort. Patients with bone metastases demonstrated comparable MTV values to those without osseous involvement (median 38.11 cm^3^ vs. 32.77 cm^3^, *p* = 0.261). Here, median MTV measures did not differ significantly.

**Figure 4:**
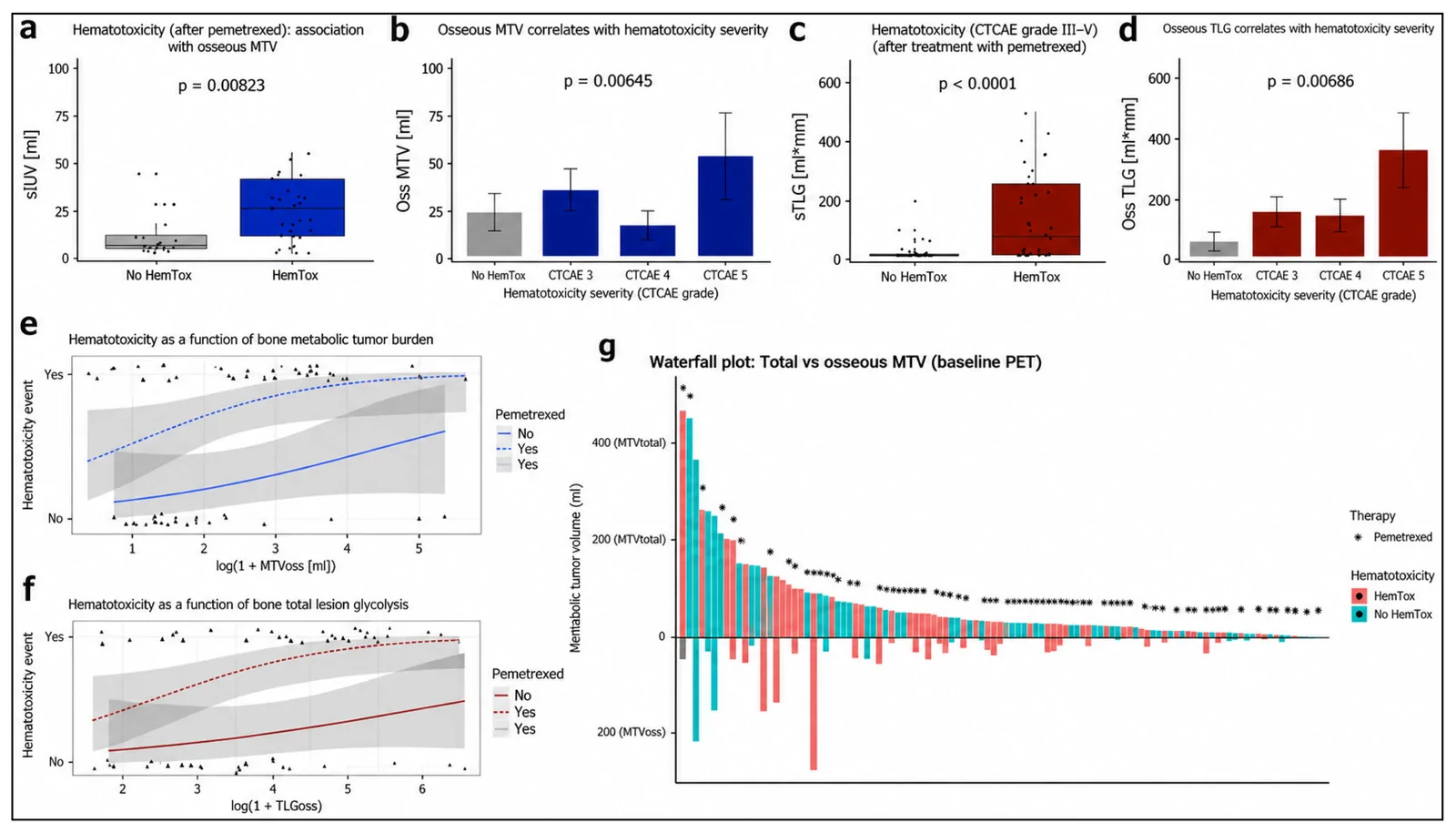
Association of osseous metabolic tumor burden with severe hematotoxicity. (**a**) Patients who developed severe hematotoxicity (CTCAE grade III–V) exhibited significantly higher baseline osseous metabolic tumor volume (MTVoss) compared with those without hematotoxicity (*p* = 0.00823). (**b**) Osseous MTV increased in parallel with the severity of hematotoxicity, demonstrating a significant correlation across CTCAE grades (*p* = 0.00645). (**c**) In patients treated with pemetrexed, higher CTCAE grades of hematotoxicity were associated with significantly increased osseous total lesion glycolysis (TLG) (*p* < 0.0001). (**d**) Likewise, osseous total lesion glycolysis (TLGoss) correlated with hematotoxicity severity (*p* = 0.00868). (**e**) Logistic regression analysis illustrates the increasing probability of severe hematotoxicity with rising osseous MTV, particularly in pemetrexed-treated patients. (**f**) A similar relationship is observed for osseous TLG, indicating a higher risk of hematotoxicity with increasing skeletal metabolic tumor burden. (**g**) Waterfall plot depicting baseline total MTV (positive bars) and osseous MTV (negative bars) on a per-patient basis, ordered by decreasing total MTV. Bars are colored according to the occurrence of hematotoxicity, and asterisks indicate pemetrexed treatment, highlighting that patients with substantial osseous tumor burden and exposure to pemetrexed are enriched. In panel (**g**), hematotoxicity indicates the occurrence of any severe hematologic toxicity (CTCAE grade III–V), irrespective of the specific hematopoietic lineage affected. In panels (**a**,**c**), black dots represent individual patient measurements. In panels (**e**,**f**), black triangles represent individual patients, indicating the presence or absence of severe hematotoxicity (CTCAE grade III–V).

When stratifying patients by treatment groups, those receiving pemetrexed had comparable median MTV to patients on non-pemetrexed regimens (33.81 cm^3^ vs. 37.66 cm^3^, *p* = 0.701). A higher osseous MTV was significantly associated with the occurrence of severe hematotoxicity (*p* = 0.00823, Figure 4a). At the same time, osseous MTV correlated with the severity of hematotoxicity (*p* = 0.00645, Figure 4b).

In addition to MTV, TLG was assessed to enhance the robustness of the evaluated parameters. Both the total TLG and, specifically, the TLG of osseous metastases were quantified. In the subgroup of patients treated with pemetrexed, osseous TLG showed a significant correlation with the occurrence of severe hematotoxicity (*p* < 0.0001, Figure 4c). Moreover, the results demonstrate that the magnitude of osseous TLG in the pemetrexed-treated subgroup correlates with the severity of the corresponding hematotoxicity (*p* = 0.00686, Figure 4d). Especially in the subgroup treated with pemetrexed, there was a strong correlation between osseous MTV (Figure 4e) and osseous TLG (Figure 4f) and the occurrence of severe hematotoxicity. Among the 108 patients with evaluable baseline PET/CT imaging, 53 experienced severe hematotoxicity events (CTCAE grade III–V). Of these, 33 patients (62.3%) demonstrated osseous metastatic involvement with a quantifiable osseous metabolic tumor volume. Notably, 27 of these patients (50.9% of all hematotoxicity events) had been treated with pemetrexed. In addition, 14 of the 53 hematotoxicity events (26.4%) occurred in patients who received pemetrexed despite the absence of detectable osseous metastases. These distributions are illustrated in Figure 4g.

These findings highlight the potential of MTV and TLG as biomarkers for assessing disease burden and predicting treatment-related toxicities in patients with osseous metastatic LUAD particularly those receiving pemetrexed.

### 3.8. Prospective Post-Implementation Cohort

Following an interim analysis of the retrospective cohort, pemetrexed-containing regimens were no longer routinely recommended for patients with bone-metastatic LUAD at our certified lung cancer center. Following implementation of this change in clinical practice, 15 consecutive patients with bone-metastatic LUAD receiving pemetrexed-free systemic treatment were prospectively observed for hematologic toxicity.

Severe hematotoxicity (CTCAE grade III–V) occurred in 9 of 15 patients (60%), comprising eight grade III and one grade V event. No grade IV hematotoxicity was observed. Hematotoxic events predominantly presented as isolated cytopenias. Febrile neutropenia occurred in three patients, three patients required red blood cell transfusions, and one hematotoxicity-related death occurred (Supplementary Table S7).

For descriptive purposes, the frequency and severity of hematotoxicity in this prospective post-implementation cohort were compared with those observed in the historical PemOss cohort. Although severe hematotoxicity remained frequent, the distribution of events in the post-implementation cohort was predominantly confined to grade III toxicity, with fewer grade IV–V events observed. Given the small sample size, non-randomized design, and comparison with a historical cohort, these observations are considered exploratory and hypothesis-generating and do not permit causal inference regarding the effect of pemetrexed avoidance.

## 4. Discussion

This study provides novel clinical evidence that pemetrexed-based chemotherapy is associated with disproportionate hematologic toxicity and inferior survival outcomes in LUAD patients with osseous metastases. The findings from this cohort of 136 patients underscore a critical interaction between the site of metastasis and treatment tolerability, identifying bone metastases as an independent risk factor for severe hematotoxicity and mortality in the context of pemetrexed therapy.

The question of whether pemetrexed induces an increased rate of severe and even fatal hematologic toxicities in patients with lung adenocarcinoma and bone metastases arose directly from daily clinical experience. In routine practice, clinicians repeatedly observed early and disproportionate marrow toxicity among patients with osseous involvement receiving pemetrexed-based chemotherapy [16,17]. The PemToxTrial was therefore designed to systematically investigate this association and to identify patient subgroups at particular risk.

Pemetrexed inhibits folate-dependent enzymes such as thymidylate synthase and dihydrofolate reductase, thereby impairing DNA synthesis and hematopoietic cell proliferation. In patients with osseous metastatic disease, reduced hematopoietic reserve related to bone marrow involvement may represent a potential biological mechanism contributing to increased susceptibility to treatment-associated hematotoxicity [4].

First, our findings demonstrate a strikingly high incidence of grade III–V hematologic adverse events among pemetrexed-treated patients with bone metastases, occurring in more than 80% of cases compared with 38% in those without skeletal involvement. Nearly half of these events emerged during the first treatment cycle, indicating an early susceptibility to hematologic toxicity in this subgroup. The predominant toxicities were anemia and neutropenia, often accompanied by thrombocytopenia and febrile neutropenia, resulting in hematotoxicity-related mortality rates exceeding 40% in the pemetrexed-treated subgroup with bone metastasis. These observations may be consistent with reduced hematopoietic reserve in patients with osseous metastatic disease, although bone marrow reserve and the extent of marrow infiltration were not directly assessed in this study [18].

Second, severe hematotoxicity was associated with poorer clinical outcomes [19]. Patients with bone metastases receiving pemetrexed had significantly shorter PFS and OS compared with the other treatment groups. Dose reductions, treatment delays, and early treatment discontinuations were also frequently observed in this subgroup. Although treatment-associated hematotoxicity may contribute to these unfavorable outcomes, the observational design of our study does not allow its effect to be separated from differences in disease burden, patient characteristics, or other prognostic factors. The observed associations therefore do not establish hematotoxicity as the causal mechanism underlying inferior survival [20].

Third, calculated multivariable models identified the presence of bone metastases as an independent predictor of severe hematotoxicity and mortality in pemetrexed-treated patients. This association remained robust after adjusting for confounders including vitamin D supplementation, proton-pump inhibitor use, phosphatase levels, bisphosphonate therapy, and the presence of other metastatic sites. Notably, neither the tumor mutational profile nor PD-L1 expression was associated with severe hematotoxicity in our cohort. These findings suggest that clinical factors related to osseous disease and potentially hematopoietic reserve may be more relevant to toxicity risk than the molecular characteristics evaluated in this study; however, the underlying biological mechanisms remain to be established.

Our study also highlights a critical gap in current evidence, as phase III LUAD trials rarely stratify patients according to metastatic site, despite growing data indicating that bone metastases markedly exacerbate chemotherapy-induced bone marrow suppression [2,21]. When contextualized within the current literature, the magnitude of hematotoxicity observed in our study is remarkable. In the pivotal KEYNOTE-189 trial, which established the efficacy of pembrolizumab plus pemetrexed and platinum chemotherapy, the reported incidence of grade ≥ 3 hematologic adverse events ranged from 18% to 47%, with anemia and neutropenia in 16% of patients [2]. Although these rates are clinically relevant, they fall far below the levels documented in our bone-metastatic cohort. This discrepancy suggests that prior trials may have underestimated marrow toxicity because they did not stratify patients by metastatic site. Indeed, skeletal involvement was neither a prespecified subgroup nor a covariate in any of the large-scale studies evaluating pemetrexed-based regimens. Emerging real-world data now support our findings. Recent analyses have independently highlighted unexpectedly high hematotoxicity rates associated with pemetrexed, particularly in heavily pretreated or elderly patients with renal impairment [22,23,24]. However, none of these studies specifically examined the impact of bone metastases as a modifying factor.

The unequal distribution of platinum backbone represents another potential treatment-related confounder, as cisplatin was more frequently administered in the pemetrexed-treated groups. However, cisplatin versus carboplatin was not associated with severe hematotoxicity in our cohort, and additional adjustment for platinum backbone did not materially alter the observed pemetrexed × bone metastasis interaction. This is consistent with randomized NSCLC data showing no excess severe anemia or neutropenia with cisplatin compared with carboplatin and a higher incidence of thrombocytopenia with carboplatin-based regimens. Nevertheless, residual confounding related to non-random treatment selection cannot be excluded.

The prospective post-implementation cohort provides additional observational context following the change in clinical practice at our center. Although severe hematotoxicity remained frequent, most events were grade III, with only one grade V event and no grade IV events observed. This pattern is consistent with a lower burden of high-grade hematotoxicity after implementation of pemetrexed-free treatment strategies. However, the small sample size, absence of randomization, and comparison with a historical cohort preclude causal inference regarding the effect of pemetrexed avoidance. These prospective observations should therefore be regarded as hypothesis-generating and require confirmation in larger, independently designed cohorts.

Clinically, these findings have relevant therapeutic implications. Our data indicate that pemetrexed is associated with a substantially increased risk of severe hematotoxicity and hematotoxicity-related mortality in LUAD patients with osseous metastases, necessitating a stringent risk–benefit evaluation, particularly in the presence of extensive skeletal involvement. Accordingly, pemetrexed-containing regimens are no longer routinely recommended for this subgroup in tumor board deliberations at the Lung Cancer Center Ulm, and alternative treatment strategies are considered on an individual basis. Prospective, independently designed studies will be required to determine whether pemetrexed avoidance translates into a clinically meaningful reduction in severe hematotoxicity.

A further limitation is the absence of systematic bone marrow aspiration or biopsy data. Bone marrow examination is not routinely performed in patients with metastatic LUAD and was available only in a small, clinically selected subset of our retrospective cohort. Consequently, radiologically detected osseous metastases cannot be equated with histologically confirmed bone marrow infiltration, and we cannot determine whether the observed susceptibility to hematotoxicity reflects direct marrow replacement by tumor cells, alterations of the hematopoietic microenvironment, reduced functional marrow reserve, or a combination of these mechanisms.

## 5. Conclusions

In conclusion, the PemToxTrial identified a strong association between pemetrexed exposure, osseous metastases, and severe hematotoxicity in patients with advanced LUAD. These findings suggest that bone metastasis status may be clinically relevant for hematotoxicity risk stratification during pemetrexed-based treatment and warrant confirmation in independent prospective studies.

## Figures and Tables

**Table 1:** Hematotoxicity profile in the study group.

Characteristics	M+ (OSS) + Pem Subgroup I (*n* = 47)	M+ (OTH) + Pem Subgroup II (*n* = 45)	M+ (OSS) No Pem Subgroup III (*n* = 22)	M+ (OTH) No Pem Subgroup IV (*n* = 22)
HemTox events	38 (81%)	17 (37%)	7 (32%)	9 (41%)
Grade III	17 (45%)	9 (53%)	6 (86%)	5 (56%)
Grade IV	8 (21%)	7 (41%)	-	3 (33%)
Grade V	13 (34%)	1 (6%)	1 (14%)	1 (11%)
Anemia	28 (74%)	12 (70%)	4 (57%)	8 (89%)
Red cell transfusions	51	21	9	18
Relative drop of hemoglobin (average %)	23	14	34	29
Thrombocytopenia	16 (42%)	3 (18%)	1 (14%)	3 (33%)
Platelets transfusions	22	1	0	2
Neutropenia	27 (71%)	12 (71%)	4 (57%)	6 (67%)
Neutropenia with fever/sepsis	15 (56%)	3 (25%)	2 (50%)	2 (33%)
Additional G-CSF	17	7	4	7
Multiple cytopenia	19 (50%)	7 (41%)	1 (14%)	6 (67%)
Timepoint of HemTox event occurrence				
Cycle 1	19 (50%)	-	3 (43%)	6 (67%)
Cycle 2	5 (13%)	2 (12%)	4 (57%)	1 (11%
Cycle 3	6 (16%)	4 (24%)	-	3 (33%)
Cycle 4	4 (11%)	6 (35%)	-	-
Cycle 5	2 (5%)	1 (6%)	-	-
Maintenance	2 (5%)	4 (24%)	-	-

G-CSF, Granulocyte-Colony Stimulating Factor; HemTox, hematotoxicity; M+ (OSS), osseous metastasis; M+ (OTH), metastasis except osseous; Pem, Pemetrexed. Percentages were calculated using the total number of patients in the respective subgroup as the denominator (PemOss, *n* = 47; PemNoOss, *n* = 45; NoPemOss, *n* = 22; NoPemNoOss, *n* = 22), unless otherwise specified.

## Data Availability

The data presented in this study are available on request from the corresponding author.

## Supplementary Materials

The following supporting information can be downloaded at: https://www.mdpi.com/article/10.3390/cancers18203281/s1, Table S1: Overview of variables of the clinicopathological data collected in the PemToxTrial; Table S2: Overview of genes analyzed using the TSO500 panel; Table S3: Concomitant treatment affecting bone metabolism; Table S4: Treatment characteristics of the study cohort; Table S5: Summary of treatment responses towards 1st line treatment; Table S6: Summary of severe adverse events (SAE) upon first line treatment; Table S7: Hematotoxicity profile in the prospective intervention cohort; Table S8: Clinicopathologic characteristics of the study cohort; Figure S1: The flow chart illustrates the composition of the study cohort in the PemTox study; Figure S2: Timeline illustrating the standardized laboratory assessment time points during (immuno-)chemotherapy in NSCLC patients at the Lung Cancer Center Ulm; Figure S3: Clinical characteristics and treatment distribution across subgroups; Figure S4: Progression-free and overall survival according to pemetrexed treatment and bone metastasis status; Figure S5: Sensitivity analyses accounting for platinum backbone; Figure S6: Genomic landscape and molecular correlates of severe hematotoxicity; Figure S7: Distribution of alteration counts across treatment subgroups; Figure S8: Association of potential driver alteration counts with hematotoxicity and progression-free survival; Figure S9: PD-L1 tumor proportion score, severe hematotoxicity, and clinical outcomes according to PD-L1 expression; Figure S10: PET/CT scan of a patient with diffuse bone metastases and elevated MTV/MTVoss (382/205 cm^3^) and TLG/TLGoss (2714/1423 SUVxcm^3^).

## Abbreviations

CTCAE	Common Terminology Criteria for Adverse Events
EANM	European Association of Nuclear Medicine
ECOG-PS	Eastern Cooperative Oncology Group Performance Status
G-CSF	Granulocyte-Colony Stimulating Factor
FFPE	Formalin-fixed, paraffin-embedded
ICH-GCP	International Conference on Harmonization Good Clinical Practice
ICI	Immune Checkpoint Inhibitors
MTV	Metabolic Tumor Volume
NGS	Next-generation sequencing
NSCLC	Non-Small Cell Lung Cancer
OS	Overall Survival
PET-CT	Positron emission tomography-computed tomography
PFS	Progression-Free Survival
PPI	Proton pump inhibitors
SUV	Standardized uptake value
TLG	Total Lesion Glycolysis
TMB	Tumor mutational burden
