# Supplementary material for: PemToxTrial: An Observational Study of PEMetrexed-Associated Severe HematoTOXicity in Lung Adenocarcinoma with Bone Metastases
Source: Cancers (Basel). 2026 Oct 12;18(20):3281. doi: 10.3390/cancers18203281 (PMC13702182; doi:10.3390/cancers18203281)
Supplement: Supplementary file 1 [file cancers-18-03281-s001.zip › cancers-4584098-supplementary.pdf]

Supplementary Material PemToxTrial

PemToxTrial: An Observational Study of PEMetrexed-Associated Severe HematoTOXicity in Lung Adenocarcinoma with Bone Metastases

Christian Riedel [1,†], Christian Neumann [2,3,†], Raban Heller [4], Arthur Bauer [2,3], Patrick Büchele [3,5], Konrad Steinestel [3,6], Michael Grunert [3,7,8], Jan Sölter [3,9], Niklas Gebauer [10], Annette Arndt [6], Matthias Müller [2,3], Armin Riecke [2,3,‡] and Hanno Witte [2,11,*,‡]

† These authors contributed equally to this work.
‡ Shared senior authorship.

Affiliations:

Please see main manuscript.

Case Identification and Data Curation

Eligible cases were identified through systematic screening of the institutional lung cancer registry and electronic medical records at the Lung Cancer Center Bundeswehrkrankenhaus Ulm. For all included patients, longitudinal clinical data were curated manually by two independent investigators and cross-validated for consistency. Extracted variables comprised baseline demographics, ECOG performance status, UICC stage, metastatic sites, treatment regimens and sequencing, laboratory values, and outcome data(1-3). In cases of ambiguous documentation, original source records were reviewed and consensus was reached within the study team.

Follow-up was performed until death or last documented clinical contact. For the prospective intervention cohort, data were collected in real time during routine visits and stored in a dedicated study database. Data cut-off dates were predefined for both cohorts.

Definition of Bone Metastatic Disease

Bone metastases were defined based on radiologic evidence from CT, PET-CT, and/or MRI at baseline staging. Lesions were classified as osseous metastases if they showed morphologic or metabolic features consistent with metastatic involvement, as assessed by board-certified radiologists and nuclear medicine physicians. Equivocal findings were reviewed in interdisciplinary consensus meetings. For patients with mixed metastatic patterns, bone involvement was coded as present if at least one skeletal lesion was identified.

Operationalization of Hematologic Toxicity Endpoints

Hematologic toxicity was operationalized according to CTCAE v6.0 definitions (4). For the purpose of statistical analyses, a severe hematotoxicity event was defined as the first occurrence of any grade III–V cytopenia (anemia, neutropenia, leukopenia, or thrombocytopenia) during systemic therapy. In patients experiencing multiple cytopenias, the highest recorded CTCAE grade was used for classification.

Composite endpoints were constructed to reflect clinically meaningful toxicity patterns:

(i) grade ≥III hematotoxicity or death,

(ii) grade ≥IV hematotoxicity or death, and

(iii) grade V hematotoxicity or death.

Deaths were attributed to hematologic toxicity if occurring in the context of severe cytopenia, febrile neutropenia, bleeding, or sepsis without alternative dominant causes.
Temporal attribution of hematotoxicity to treatment was ensured by restricting events to those occurring during active systemic therapy or within 30 days after the last administration.

Handling of Laboratory Data and Longitudinal Measures

Baseline laboratory values were defined as the last measurements obtained within 7 days prior to initiation of systemic therapy. On-treatment nadir values were identified for each blood parameter across all available measurements during therapy. For longitudinal visualization of hemoglobin trends, values were aligned to treatment cycles and interpolated if necessary.
In cases of missing intermediate laboratory values, no imputation was performed. Analyses were based on available data only. Consistency checks were applied to detect implausible values, which were verified against source records.

Details of Chemotherapy Exposure and Supportive Care

Exposure to pemetrexed was defined as receipt of at least one cycle of a pemetrexed-containing regimen. Dosing schedules, combination partners (e.g., platinum compounds, immune checkpoint inhibitors), and maintenance strategies were recorded in detail. Supportive measures including vitamin supplementation, transfusions, and G-CSF administration were extracted to contextualize hematologic events and to characterize management strategies.
Dose modifications were categorized as reductions, delays, or permanent discontinuations and linked temporally to hematotoxicity events when applicable.

Extended Description of Molecular Data Generation

All analyses were conducted using formalin-fixed, paraffin-embedded (FFPE) tissue samples. DNA extraction from tumor tissue was performed using the Maxwell® CSC DNA FFPE kits on the Maxwell® CSC instrument, in accordance with the manufacturer's instructions (Promega, Madison, WI, USA). The concentration of isolated nucleic acids was determined fluorometrically using the Qubit 4 fluorometer (Thermo Fisher Scientific, Waltham, MA, USA). For DNA-based targeted next-generation sequencing (NGS), the TruSight Oncology 500 kit (San Diego, CA, USA) was employed. A total of 100 ng of genomic DNA was fragmented using an ME 220 focused ultrasonicator (Brighton, UK). In cases were 100 ng DNA was not reached the maximum input volume of 12 µl was used. DNA library preparation was performed according to the manufacturer's protocol. Quantification, quality control, normalization, and

pooling of the libraries followed the guidelines provided in the TruSight Oncology 500 protocol. NGS was subsequently performed by loading 1 pM of pooled libraries onto the NextSeq 550Dx sequencer (San Diego, CA, USA).

Data processing

Primary and secondary analyses of the sequencing data, including the generation of FASTQ files, read alignment to the hg19 reference genome, and the generation of VCF files, were performed using the onboard TruSight Oncology 500 v2.2 Local App (Illumina, San Diego, CA, USA). For variant classification, the files generated from DNA sequencing—such as the small variants file (.genome.vcf), copy number variants file (.vcf), microsatellite instability (MSI) file (.msi.json), and tumor mutational burden (TMB) file (.tmb.json)—were converted to the Molecular Health file format using the MH VCF Adapter Suite version 2.1.0. The resulting files were subsequently uploaded to the Molecular Health Guide database platform (version 5.3.0) (Molecular Health GmbH, Heidelberg, Germany) for further analysis.

PET-CT Segmentation Workflow and Quality Control Using LIFEx

Baseline 18F-FDG PET-CT images were retrieved in DICOM format from the institutional picture archiving and communication system and analyzed using LIFEx radiomics software (version 25.06.1)(5). All scans had been acquired according to institutional PET protocols aligned with EANM (European Association of Nuclear Medicine) recommendations, including standardized patient preparation, uptake times, and reconstruction settings(6).

Images were imported into LIFEx and resampled to isotropic voxel sizes using the software's default interpolation algorithm to ensure comparability across scans. Lesion segmentation was performed using a semi-automatic workflow. After manual initialization of regions of interest around visually apparent hypermetabolic lesions, a relative threshold of >40% of the lesion-specific SUVmax was applied to delineate metabolically active tumor tissue. This approach was chosen to balance sensitivity and robustness across lesions with heterogeneous uptake patterns.

Physiological FDG uptake in organs such as brain, myocardium, liver, bowel, kidneys, urinary tract, and sites of known inflammatory activity was carefully excluded by manual editing. For lesions adjacent to high physiological background activity, segmentation boundaries were refined manually to avoid spill-in effects.

All hypermetabolic lesions were segmented individually. Metabolic tumor volume (MTV) was calculated automatically by LIFEx as the sum of all voxels within the segmented volumes

exceeding the SUV threshold. Total MTV was defined as the cumulative volume of all segmented lesions per patient. Osseous MTV (MTVoss) was derived by summing the MTV of lesions localized to the skeletal system only. Lesion classification as osseous was based on anatomical CT correlation and radiologic reports.

In addition to MTV, total lesion glycolysis (TLG) was calculated for each lesion as the product of MTV and the mean SUV within the segmented volume. Total TLG and osseous TLG (TLGoss) were obtained by summation across all lesions or bone lesions, respectively, and served as complementary metrics of metabolic tumor burden.

Segmentation and quantification were independently performed by two trained investigators blinded to clinical outcomes and treatment allocation, under the supervision of a board-certified nuclear medicine physician (MG). Inter-observer agreement was assessed by comparing total MTV and MTVoss values between both readers; the absolute inter-observer variability was <1%. In cases with discrepancies exceeding 5% at the lesion or patient level, segmentations were reviewed jointly and a consensus segmentation was generated.

All segmentations underwent visual quality control to ensure accurate lesion delineation and exclusion of non-tumoral uptake. MTV and TLG values were exported from LIFEx and integrated into the clinical database for downstream statistical analyses. Only volumetric and first-order metabolic parameters were used for analysis to ensure methodological robustness and clinical interpretability, and no higher-order texture features were considered.

<u>Radiomics Feature Handling</u>

Although LIFEx allows extraction of multiple radiomic features, only volumetric and first-order metabolic parameters (MTV and TLG) were used in this study to ensure robustness and clinical interpretability. No higher-order texture features were included to avoid overfitting given the cohort size.

<u>Statistical Modeling and Sensitivity Analyses</u>

For correlation analyses between imaging biomarkers and hematotoxicity severity, non-parametric trend tests and ordinal logistic regression models were applied. MTV and TLG were analyzed both as continuous variables and after log-transformation to account for right-skewed distributions. For regression analyses, standardized z-scores were used to allow comparison of effect sizes.

Sensitivity analyses were performed excluding patients with baseline cytopenias, prior radiotherapy to large bone volumes, or early treatment discontinuation (<2 cycles) to assess the robustness of the observed associations.

To formally assess whether bone metastasis status modified the association between pemetrexed exposure and severe hematotoxicity, logistic regression models included pemetrexed exposure, bone metastasis status, and a multiplicative pemetrexed × bone metastasis interaction term. Because the distribution of platinum compounds differed between treatment subgroups, platinum backbone (cisplatin vs. carboplatin) was additionally evaluated as a potential treatment-related confounder. Among patients receiving platinum-based therapy, cisplatin versus carboplatin was not associated with severe hematotoxicity in univariable analysis (OR = 1.05, 95% CI 0.53–2.11, p = 0.883). Addition of platinum backbone to the interaction model did not materially alter the pemetrexed × bone metastasis interaction (interaction OR = 12.07, 95% CI 2.40–60.59, p = 0.0025), while platinum type itself was not significantly associated with severe hematotoxicity (OR = 0.61, 95% CI 0.26–1.43, p = 0.254). In a further sensitivity analysis additionally adjusting for age, ECOG performance status, proton-pump inhibitor use, vitamin D supplementation, bisphosphonate therapy, alkaline phosphatase levels, and additional metastatic sites, the interaction remained statistically significant (adjusted interaction OR = 6.58, 95% CI 1.01–42.97, p = 0.049), whereas platinum backbone remained unrelated to severe hematotoxicity (adjusted OR = 1.18, 95% CI 0.39–3.53, p = 0.771). The corresponding sensitivity analyses are presented in **Supplementary Figure 5.**

The subsequent prospective post-implementation cohort was analyzed descriptively. Given its small sample size, non-randomized design, and comparison with a historical cohort, this analysis was considered exploratory and hypothesis-generating. No comparative treatment-effect estimates, relative or absolute risk reductions, or numbers needed to treat or harm were calculated, and no causal inference regarding the effect of pemetrexed avoidance was made.

No formal adjustment for multiple testing was applied, and all secondary and exploratory analyses were considered hypothesis-generating.

<u>Software and Reproducibility</u>

All statistical analyses were scripted and version-controlled. Key analyses were independently reproduced by a second investigator to ensure reproducibility. Imaging analyses were performed using LIFEx v25.06.1, and molecular analyses used the respective manufacturer-provided pipelines. Graphical outputs were generated using R (v4.5.2) and BioRender (Toronto, Canada).

Ethical and Data Governance Considerations

All data were handled in accordance with local data protection regulations. Only de-identified datasets were used for analysis. Access to source data was restricted to authorized study personnel. The prospective cohort was embedded into routine clinical workflows and did not require protocol-driven interventions beyond observational data collection. The study was conducted in compliance with the Declaration of Helsinki and the International Conference on Harmonization Good Clinical Practice (ICH-GCP) guidelines(7). Ethics approval was obtained from the University of Ulm Ethics Committee (reference no. 112-23).

Supplementary Results

Molecular Characteristics of the Cohort (Comprehensive Results)

Molecular profiling using NGS was performed on tumor samples from 90 patients to characterize key genomic alterations in the NSCLC cohort (**Supplementary Figure S6a**). The most frequent mutations involved *KRAS* (38%) and *TP53* (29%). The broader mutational spectrum reflected canonical alterations typical of lung adenocarcinoma, including *STK11* (11%), *CDKN2A* (5%), and *KEAP1* (4%) mutations. Subgroup-specific mutational patterns are detailed in **Supplementary Figure S6a**. Despite comprehensive analysis, none of the twelve most frequently mutated genes were significantly associated with high-grade hematotoxicity, suggesting that toxicity patterns were independent of the tumor's genomic profile (**Supplementary Figure S6b**).

The prevalence of molecular alterations was comparable between pemetrexed-treated and non-pemetrexed patients, and mutation frequencies did not differ between cases with or without severe hematotoxicity. Among *KRAS* mutant cases, *KRAS G12C* represented the predominant variant (56%).

The number of potential driver alterations per case was also assessed (**Supplementary Figure S7)**. Univariate analysis evaluated their impact on severe hematotoxicity and PFS (**Supplementary Figures S8a & b**). A statistical trend indicated that patients harboring four or more potential driver alterations had inferior PFS, whereas the number of alterations showed no significant association with severe hematotoxic events.

Programmed death-ligand 1 (**PD-L1**) expression was assessed by immunohistochemistry in all samples. High PD-L1 expression (TPS $\geq$ 50%) was observed in 27% (n = 36) of patients,

intermediate expression (1–49%) in 38% (n = 51), and low expression (< 1%) in 36% (n = 49). PD-L1 expression did not correlate significantly with severe hematotoxicity in the pemetrexed-treated subgroup (p = 0.977). The distribution of PD-L1 expression levels between the four subgroups is shown in **Supplementary Figure S9** and summarized in **Supplementary Table S8** displaying a higher median for TPS measures in the non-pemetrexed treated subgroup.

Supplementary Table S1. Overview of variables of the clinicopathological data collected in the PemToxTrial

Category	Variables		
Clinical features	Sex	Body-Mass-Index	ECOG
	Age at diagnosis	Primary location	B-Symptoms
	Date of initial diagnosis	Stadium UICC8	Secondary tumor
	Size	TNM	PET-CT available
	Weight	Presence of Ascites	Date 1st PET-CT
	Smoking status	Presence of pleural effusions	
Treatment	Dose Pemetrexed	2nd line cycles	Maintenance therapy
	Date start Pemetrexed	3rd line Chemotherapy	Medication with Proton pump inhibitor
	Dose Carbo-/Cisplatin	3rd line cycles	Medication with Bisphosphonates
	Immuntherapy	4th line Chemotherapy	Medication with Vitamin D
	Targeted therapy	4th line cycles	Medication with Allopurinol/Febuxostat
	1st line Chemotherapy	5th line Chemotherapy	Antibiotic therapy before Hematotoxicity
	1st line cycles	5th line cycles	Therapy response
	2nd line Chemotherapy		
Laboratory results	Albumin during diagnosis	AST during diagnosis	Hemoglobin in Hematotoxicity
	Total-Calcium during diagnosis	ALT during diagnosis	Hematocrit in Hematotoxicity
	CRP during diagnosis	Bilirubin during diagnosis	Erythrocyte count in Hematotoxicity
	WBC during diagnosis	Level of Folic acid during diagnosis	MCV in Hematotoxicity
	Hemoglobin during diagnosis	Level of Vitamin B12 during diagnosis	LDH in Hematotoxicity
	Hematocrit during diagnosis	Hemoglobin before 2nd cycle	Alkaline phosphatase in Hematoxicity
	Erythrocyte count during diagnosis	Decrease in Hemoglobin before 2nd cycle (total and relative)	GGT in Hematoxicity
	MCV during diagnosis	Albumin in Hematotoxicity	AST in Hematoxicity
	LDH during diagnosis	CRP in Hematotoxicity	ALT in Hematoxicity
	Alkaline phosphatase during diagnosis	WBC in Hematotoxicity	Bilirubin in Hematoxicity
	GGT during diagnosis	Neutrophils in Hematotoxicity	Normoblasts in Hematoxicity
Events	Hematoxicitiy during Pemetrexed	Count of Thrombocyte concentrate	Progression-free Survival
	Date Hematotoxicity	Duration of Nadir	Date of Death
	Days till Hematotoxicity	Additional GCS-F before Hematoxicity	Death Event due to hematoxicity
	Cycle of chemotherapy during Hematotoxicity	Toxicity besides Hematoxicity	Overall Survival (since diagnosis)
	Grade of Hematotoxicity	Relapse	Overall Survival (since start of Pemetrexed)
	Type of Hematotoxicity	Event-free Survival	Last contact
	Fatal Event (Death/ICU)	Date of progression	Observation time
	Count of Erythrocyte concentrate		
histological findings	Histology	TPS	NGS Panel available
	Ki67	CPS	kind of mutation

	TTF-1	IC	Tumor Mutational Burden
	Napsin		

Supplementary Table S2. Overview of genes analyzed using the TSO500 panel.

ABL1	CD79B	ERCC5	GNAQ	KDM6A	NF2	PRKCI	SNCAIP
ABL2	CDC73	ERG	GNAS	KDR	NFE2L2	PRKDC	SOCS1
ACVR1	CDH1	ERRFI1	GPR124	KEAP1	NFKBIA	PRSS8	SOX10
ACVR1B	CDK12	ESR1	GPS2	KEL	NKX2-1	PTCH1	SOX17
AKT1	CDK4	ETS1	GREM1	KIF5B	NKX3-1	PTEN	SOX2
AKT2	CDK6	ETV1	GRIN2A	KIT	NOTCH1	PTPN11	SOX9
AKT3	CDK8	ETV4	GRM3	KLF4	NOTCH2	PTPRD	SPEN
ALK	CDKN1A	ETV5	GSK3B	KLHL6	NOTCH3	PTPRS	SPOP
ALOX12B	CDKN1B	ETV6	H3F3A	KMT2B	NOTCH4	PTPRT	SPTA1
ANKRD11	CDKN2A	EWSR1	H3F3B	KMT2C	NPM1	QKI	SRC
ANKRD26	CDKN2B	EZH2	H3F3C	KMT2D	NRAS	RAB35	SRSF2
APC	CDKN2C	FAM123B	HGF	KRAS	NRG1	RAC1	STAG1
AR	CEBPA	FAM175A	HIST1H1C	LAMP1	NSD1	RAD21	STAG2
ARAF	CENPA	FAM46C	HIST1H2BD	LATS1	NTRK1	RAD50	STAT3
ARFRP1	CHD2	FANCA	HIST1H3A	LATS2	NTRK2	RAD51	STAT4
ARID1A	CHD4	FANCC	HIST1H3B	LMO1	NTRK3	RAD51B	STAT5A
ARID1B	CHEK1	FANCD2	HIST1H3D	LRP1B	NUP93	RAD51C	STAT5B
ARID2	CHEK2	FANCE	HIST1H3E	LYN	NUTM1	RAD51D	STK11
ARID5B	CIC	FANCF	HIST1H3F	LZTR1	PAK1	RAD52	STK40
ASXL1	CREBBP	FANCG	HIST1H3G	MAGI2	PAK3	RAD54L	SUFU
ASXL2	CRKL	FANCI	HIST1H3H	MALT1	PAK7	RAF1	SUZ12
ATM	CRLF2	FANCL	HIST1H3I	MAP2K1	PALB2	RANBP2	SYK
ATR	CSF1R	FAS	HIST1H3J	MAP2K2	PARK2	RARA	TAF1
ATRX	CSF3R	FAT1	HIST2H3A	MAP2K4	PARP1	RASA1	TBX3
AURKA	CSNK1A1	FBXW7	HIST2H3C	MAP3K1	PAX3	RB1	TCEB1
AURKB	CTCF	FGF1	HIST2H3D	MAP3K13	PAX5	RBM10	TCF3
AXIN1	CTLA4	FGF10	HIST3H3	MAP3K14	PAX7	RECQL4	TCF7L2
AXIN2	CTNNA1	FGF14	HLA-A	MAP3K4	PAX8	REL	TERC
AXL	CTNNB1	FGF19	HLA-B	MAPK1	PBRM1	RET	TERTb
B2M	CUL3	FGF2	HLA-C	MAPK3	PDCD1	RFWD2	TET1
BAP1	CUX1	FGF23	HNF1A	MAX	PDCD1LG2	RHEB	TET2
BARD1	CXCR4	FGF3	HNRNPK	MCL1	PDGFRA	RHOA	TFE3
BBC3	CYLD	FGF4	HOXB13	MDC1	PDGFRB	RICTOR	TFRC
BCL10	DAXX	FGF5	HRAS	MDM2	PDK1	RIT1	TGFBR1
BCL2	DCUN1D1	FGF6	HSD3B1	MDM4	PDPK1	RNF43	TGFBR2
BCL2L1	DDR2	FGF7	HSP90AA1	MED12	PGR	ROS1	TMEM127
BCL2L11	DDX41	FGF8	ICOSLG	MEF2B	PHF6	RPS6KA4	TMPRSS2
BCL2L2	DHX15	FGF9	ID3	MEN1	PHOX2B	RPS6KB1	TNFAIP3
BCL6	DICER1	FGFR1	IDH1	MET	PIK3C2B	RPS6KB2	TNFRSF14
BCOR	DIS3	FGFR2	IDH2	MGA	PIK3C2G	RPTOR	TOP1
BCORL1	DNAJB1	FGFR3	IFNGR1	MITF	PIK3C3	RUNX1	TOP2A
BCR	DNMT1	FGFR4	IGF1	MLH1	PIK3CA	RUNX1T1	TP53
BIRC3	DNMT3A	FH	IGF1R	MLL	PIK3CB	RYBP	TP63
BLM	DNMT3B	FLCN	IGF2	MLLT3	PIK3CD	SDHA	TRAF2
BMPR1A	DOT1L	FLI1	IKBKE	MPL	PIK3CG	SDHAF2	TRAF7

BRAF	E2F3	FLT1	IKZF1	MRE11A	PIK3R1	SDHB	TSC1
BRCA1a	EED	FLT3	IL10	MSH2	PIK3R2	SDHC	TSC2
BRCA2a	EGFL7	FLT4	IL7R	MSH3	PIK3R3	SDHD	TSHR
BRD4	EGFR	FOXA1	INHA	MSH6	PIM1	SETBP1	U2AF1
BRIP1	EIF1AX	FOXL2	INHBA	MST1	PLCG2	SETD2	VEGFA
BTG1	EIF4A2	FOXO1	INPP4A	MST1R	PLK2	SF3B1	VHL
BTK	EIF4E	FOXP1	INPP4B	MTOR	PMAIP1	SH2B3	VTCN1
C11orf30	EML4	FRS2	INSR	MUTYH	PMS1	SH2D1A	WISP3
CALR	EP300	FUBP1	IRF2	MYB	PMS2	SHQ1	WT1
CARD11	EPCAM	FYN	IRF4	MYC	PNRC1	SLIT2	XIAP
CASP8	EPHA3	GABRA6	IRS1	MYCL1	POLD1	SLX4	XPO1
CBFB	EPHA5	GATA1	IRS2	MYCN	POLE	SMAD2	XRCC2
CBL	EPHA7	GATA2	IST1H3C	MYD88	PPARG	SMAD3	YAP1
CCND1	EPHB1	GATA3	JAK1	MYOD1	PPM1D	SMAD4	YES1
CCND2	ERBB2	GATA4	JAK2	NAB2	PPP2R1A	SMARCA4	ZBTB2
CCND3	ERBB3	GATA6	JAK3	NBN	PPP2R2A	SMARCB1	ZBTB7A
CCNE1	ERBB4	GEN1	JUN	NCOA3	PPP6C	SMARCD1	ZFHX3
CD274	ERCC1	GID4	KAT6A	NCOR1	PRDM1	SMC1A	ZNF217
CD276	ERCC2	GLI1	KDM5A	NEGR1	PREX2	SMC3	ZNF703
CD74	ERCC3	GNA11	KDM5C	NF1	PRKAR1A	SMO	ZRSR2
CD79A	ERCC4	GNA13					

Supplementary Table S3. Concomitant treatment affecting bone metabolism.

Responses	M+ (OSS) + Pem Subgroup I (n = 47)	M+ (OTH) + Pem Subgroup II (n = 45)	M+ (OSS) No Pem Subgroup III (n = 22)	M+ (OTH) No Pem Subgroup IV (n = 22)
Vitamin D supplementation	7 (15%)	7 (16%)	3 (14%)	3 (14%)
Proton pump inhibitors	45 (96%)	42 (93%)	19 (86%)	20 (91%)
Bisphosphonates	16 (34%)	1 (2%)	11 (50%)	1 (5%)

M+(OSS), osseous metastasis; M+(OTH), metastasis except osseous

Supplementary Table S4. Treatment characteristics of the study cohort.

Characteristics	M+ (OSS) + Pem Subgroup I (n = 47)	M+ (OTH) + Pem Subgroup II (n = 45)	M+ (OSS) No Pem Subgroup III (n = 22)	M+ (OTH) No Pem Subgroup IV (n = 22)
**1st line treatment**				
IMPower-130	-	-	2 (9%)	1 (5%)
IMpower-150	-	-	8 (36%)	3 (14%)
KEYNOTE-189	32 (68%)	28 (62%)	-	-
KEYNOTE-407	-	-	1 (5%)	1 (5%)
CHECKMATE-012	-	-	-	1 (5%)
CHECKMATE-9LA	-	-	3 (14%)	6 (27%)
POSEIDON	2 (4%)	3 (7%)	4 (18%)	2 (9%)
BEVERLY	-	-	-	1 (5%)
Pointbreak	1 (2%)	1 (2%)	-	-
AVAil	-	-	-	1 (5%)
EMPOWER Lung-3	-	-	1 (5%)	-
Platinum doublet	12 (26%)	13 (29%)	1 (5%)	5 (23%)
ICI mono	-	-	2 (5%)	1 (5%)
**Immune checkpoint Inhibitor**				
Atezolizumab	-	-	10 (48%)	4 (27%)
Nivolumab	-	-	-	1 (7%)
Nivolumab/Ipilimumab	-	-	3 (14%)	6 (4%)
Pembrolizumab	32 (94%)	28 (90%)	3 (14%)	2 (13%)
Cemiplimab	-	-	1 (5%)	-
Durvalumab/Tremelimumab	2 (6%)	3 (10%)	4 19%)	2 (13%)
**Dose reduction platinum**				
**Yes**	17 (36%)	18 (40%)	10 (50%)	16 (80%)
**No**	30 (64%)	27 (60%)	10 (50%)	4 (20%)
**Dose reduction pemetrexed**				
**Yes**	3 (6%)	3 (7%)	-	-
**No**	44 (94%)	42 (93%)	-	-
**2nd line treatment**				
	n=18	n=16	n=6	n=4
**Targeted treatment**				
- Crizotinib	1 (6%)	1 (6%)	-	-
- Selpercatinib	1 (6%)	-	-	-
- Sotorasib	-	1 (6%)	1 (17%)	-
- Osimertinib	-	-	2 (33%)	-
**Chemotherapy only**				
- Mono	2 (11%)	2 (13%)	-	3 (75%)
- Combination	1 (6%)	1 (6%)	1 (17%)	-
**Immune checkpoint inhibition**	-	1 (6%)	1 (17%)	1 (25%)
**Nintendanib/Docetaxel**	5 (28%)	4 (25%)	1 (17%)	-
**Ramucirumab/Docetaxel**	8 (44%)	6 (38%)	-	-
**3rd line treatment**				
**Targeted treatment**	n=6	n=5	n=2	n=0
- Brigatinib	-	1 (20%)	-	-
- Afatinib	-	1 (20%)	-	-
- Sotorasib	1 (17%)	-	-	-
**Chemotherapy only**				
- Mono	3 (50%)	1 (20%)	1 (50%)	-
**Immune checkpoint inhibition**	2 (33%)	2 (40%)	-	-
**Ramucirumab/Docetaxel**	-	-	1 (50%)	-

M+(OSS), osseous metastasis; M+(OTH), metastasis except osseous

Supplementary Table S5. Summary of treatment responses towards 1st line treatment.

Responses	M+ (OSS) + Pem Subgroup I (n = 47)	M+ (OTH) + Pem Subgroup II (n = 45)	M+ (OSS) No Pem Subgroup III (n = 22)	M+ (OTH) No Pem Subgroup IV (n = 22)
Complete remission	-	1 (2%)	-	-
Partial remission	17 (36%)	23 (51%)	8 (36%)	12 (55%)
Stable disease	13 (28%)	15 (33%)	5 (23%)	6 (27%)
Refractory disease	9 (19%)	5 (11%)	5 (23%)	2 (9%)
Death rate	31 (66%)	11 (24%)	6 (27%)	9 (41%)

M+(OSS), osseous metastasis; M+(OTH), metastasis except osseous

Supplementary Table S6. Summary of severe adverse events (SAE) upon first line treatment.

**Severe Adverse events SAE grade ≥3**	**M+ (OSS) + Pem Subgroup I (n = 47)**	**M+ (OTH) + Pem Subgroup II (n = 45)**	**M+ (OSS) No Pem Subgroup III (n = 22)**	**M+ (OTH) No Pem Subgroup IV (n = 22)**
Elevated liver enzymes	-	1 (2%)	-	1 (4%)
Mucositis	6 (13%)	5 (11%)	-	-
Emesis	1 (2%)	-	-	-
Hypokalemia	1 (2%)	-	-	-
Hyperkalemia	1 (2%)	-	-	-
Acute kidney injury	5 (11%)	-	2 (9%)	-
Dizziness	1 (2%)	1 (2%)	-	-
Pneumonia/Pneumonitis	3 (6%)	-	-	1 (4%)
Colitis/diarrhea	2 (4%)	-	1 (4%)	3 (14%)
Hypertension	-	-	1 (4%)	-
Pericardial effusion	-	-	1 (4%)	-
Cutaneous toxicity/allergic reaction	-	1 (2%)	2 (9%)	-

M+(OSS), osseous metastasis; M+(OTH), metastasis except osseous

Supplementary Table S7. Hematotoxicity profile in the prospective intervention cohort.

Characteristics	M+ (OSS) n=15
HemTox events	9 (60%)
Grade III	8 (89%)
Grade IV	-
Grade V	1 (11%)
Anemia	6 (67%)
Red cell transfusions	3
Thrombocytopenia	-
Platelets transfusions	-
Neutropenia	3 (33%)
Neutropenia with fever/sepsis	3 (100%)
Additional G-CSF	3 (100%)
Multiple cytopenia	-
Fatal HemTox events	1
Cycle 1	2
Cycle 2	3
Cycle 3	3
Cycle 4	-
Cycle 5	-
Maintenance	-

HemTox, hematotoxicity; M+(OSS), osseous metastasis

Supplementary Table S8. Clinicopathologic characteristics of the study cohort.

Characteristics	M+ (OSS) + Pem Subgroup I (n = 47)	M+ (OTH) + Pem Subgroup II (n = 45)	M+ (OSS) No Pem Subgroup III (n = 22)	M+ (OTH) No Pem Subgroup IV (n = 22)
**Age (median, range)**	67 (49 - 88)	66 (47 - 81)	67 (36 - 84)	66 (43 - 84)
**Male**	30 (64%)	27 (60%)	14 (64%)	10 (45%)
**Female**	17 (36%)	18 (40%)	8 (36%)	12 (55%)
**Smoking status**				
- Never	12 (26%)	4 (9%)	4 (18%)	4 (18%)
- Former	17 (36%)	14 (31%)	11 (50%)	7 (32%)
- Current	18 (38%)	27 (60%)	7 (32%)	11 (50%)
**Packyears**				
- Median (range)	25 (2 – 70)	25 (2 – 150)	20 (8 – 90)	20 (13 – 60)
**Primary localization**				
- Right/left	27 (57%)/18 (38%)	28 (62%)/15 (33%)	16 (73%)/4 (18%)	12 (55%)/8 (36%)
- Upper lobe	10 (21%)	28 (62%)	7 (32%)	11 (50%)
- Middle lobe	2 (4%)	2 (4%)	1 (5%)	1 (5%)
- Lower lobe	12 (26%)	10 (22%)	8 (36%)	4 (18%)
- Hilar region	5 (11%)	3 (7%)	4 (18%)	4 (18%)
- Diffuse	1 (2%)	2 (4%)	1 (5%)	2 (9%)
**Localization of metastasis**				
- Brain	12 (26%)	18 (40%)	4 (18%)	7 (32%)
- Liver	12 (26%)	3 (7%)	8 (36%)	3 (14%)
- Bones	47 (100%)	-	22 (100%)	-
- Adrenal	13 (28%)	8 (18%)	4 (18%)	4 (18%)
- Pleura	15 (32%)	12 (27%)	3 (14%)	5 (23%)
- Lung	13 (28%)	15 (33)	3 (14%)	6 (27%)
- Soft tissue	1 (2%)	1 (22%)	1 (5%)	1 (5%)
- N1	3 (6%)	5 (11%)	1 (5%)	3 (14%)
- N2	10 (21%)	9 (20%)	4 (18%)	2 (9%)
- N3	23 (49%)	19 (42%)	13 (59%)	15 (68%)
- Others	8 (17%)	4 (9%)	4 (18%)	7 (32%)
**Tumor expansion**				
- T 1 – 2	10 (21%)	13 (28%)	6 (27%)	6 (27%)
- T 3 – 4	29 (62%)	26 (58%)	13 (59%)	14 (64%)
**ECOG-PS**				
- 0 – 1	38 (81%)	42 (89%)	20 (91%)	19 (86%)
- ≥2	9 (19%)	3 (11%)	2 (9%)	3 (14%)
**B-symptoms**				
- No	27 (57%)	29 (64%)	12 (55%)	11 (50%)
- Yes	20 (43%)	16 (36%)	10 (45%)	11 (50%)
**Lactate dehydrogenase**	n=46	n=44	n=22	n=22
- ≤ 240 U/l (≤4µkat/l)	22 (48%)	31 (72%)	9 (41%)	14 (64%)
- > 240 U/l (>4µkat/l)	24 (52%)	12 (28%)	13 (59%)	8 (36%)
**Lines of therapy**				
- 1	29 (62%)	29 (64%)	16 (72%)	18 (82%)
- 2	12 (26%)	11 (24%)	4 (18%)	4 (18%)
- ≥3	6 (13%)	5 (11%)	2 (9%)	-
**PD-L1 Expression TPS**				
- 0	5 (11%)	5 (11%)	3 (15%)	7 (33%)
- 1 – 49%	29 (61%)	26 (58%)	12 (54%)	10 (44%)
- ≥ 50%	13 (28%)	14 (31%)	7 (31%)	5 (23%)
**TTF1 status**	n=43	n=40	n=22	n=21
- **Negative**	13 (30%)	7 (18%)	7 (32%)	13 (62%)
- **Positive**	30 (70%)	33 (83%)	15 (68%)	8 (38%)

ECOG-PS, Eastern Cooperatice Oncology Group Performance Status; M+(OSS), osseous metastasis; M+(OTH), metastasis except osseous; PD-L1, Programmed Death-Ligand 1; Pem, Pemetrexed; TPS, Tumor Proportion Score; TTF1, Thyroid Transcription Factor-1

Supplementary Figure legends

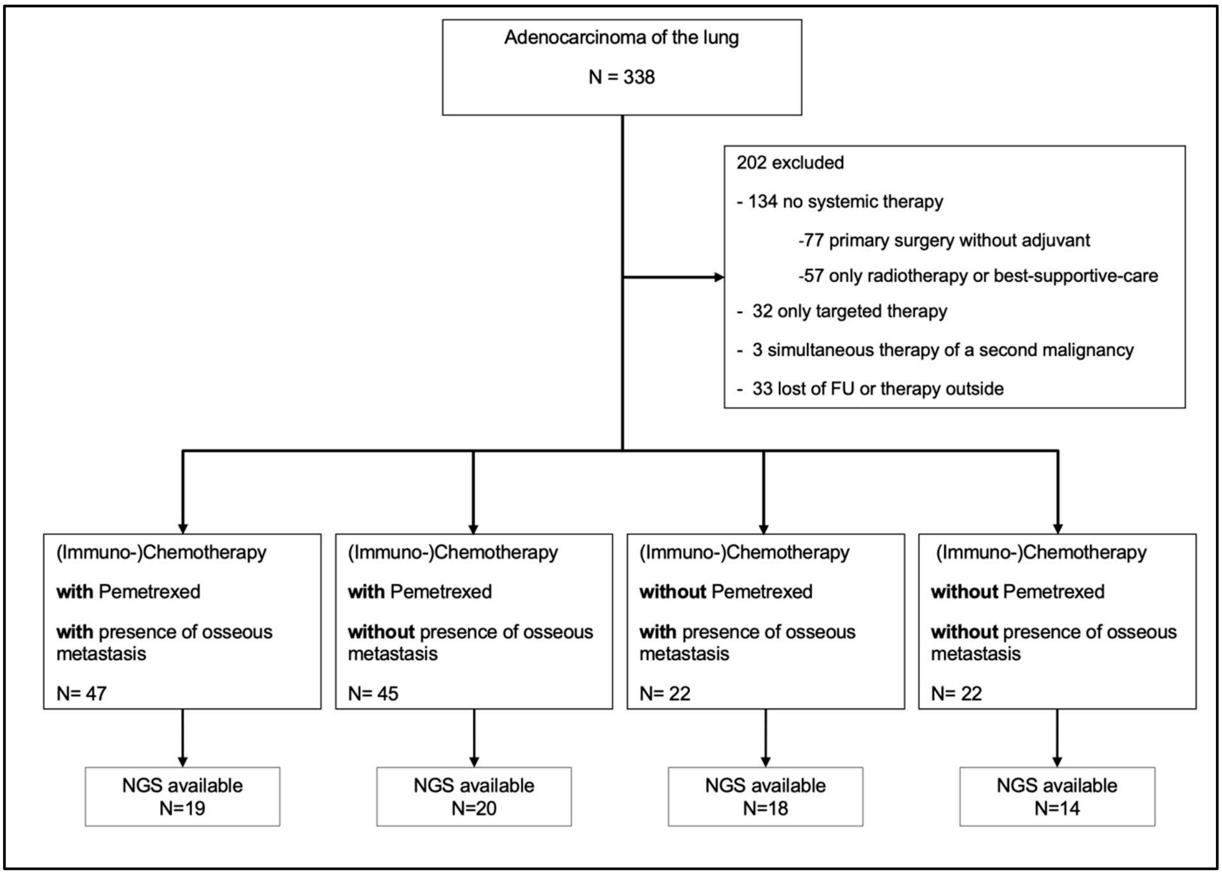

Supplementary Figure S1. The flow chart illustrates the composition of the study cohort in the PemTox study.

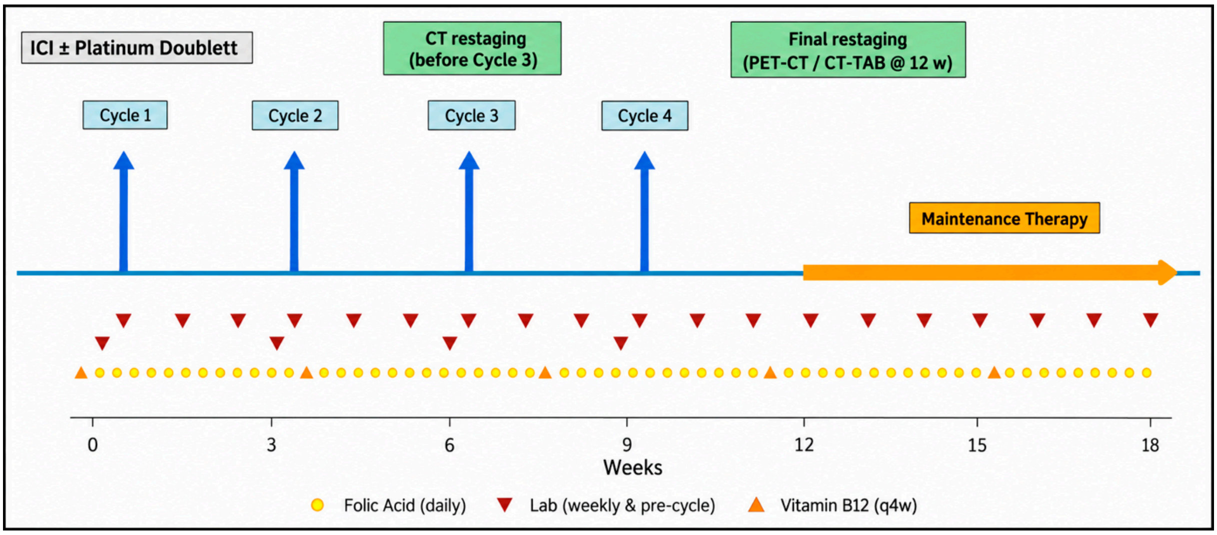

Supplementary Figure S2. Timeline illustrating the standardized laboratory assessment time points during (immuno-)chemotherapy in NSCLC patients at the Lung Cancer Center Ulm.

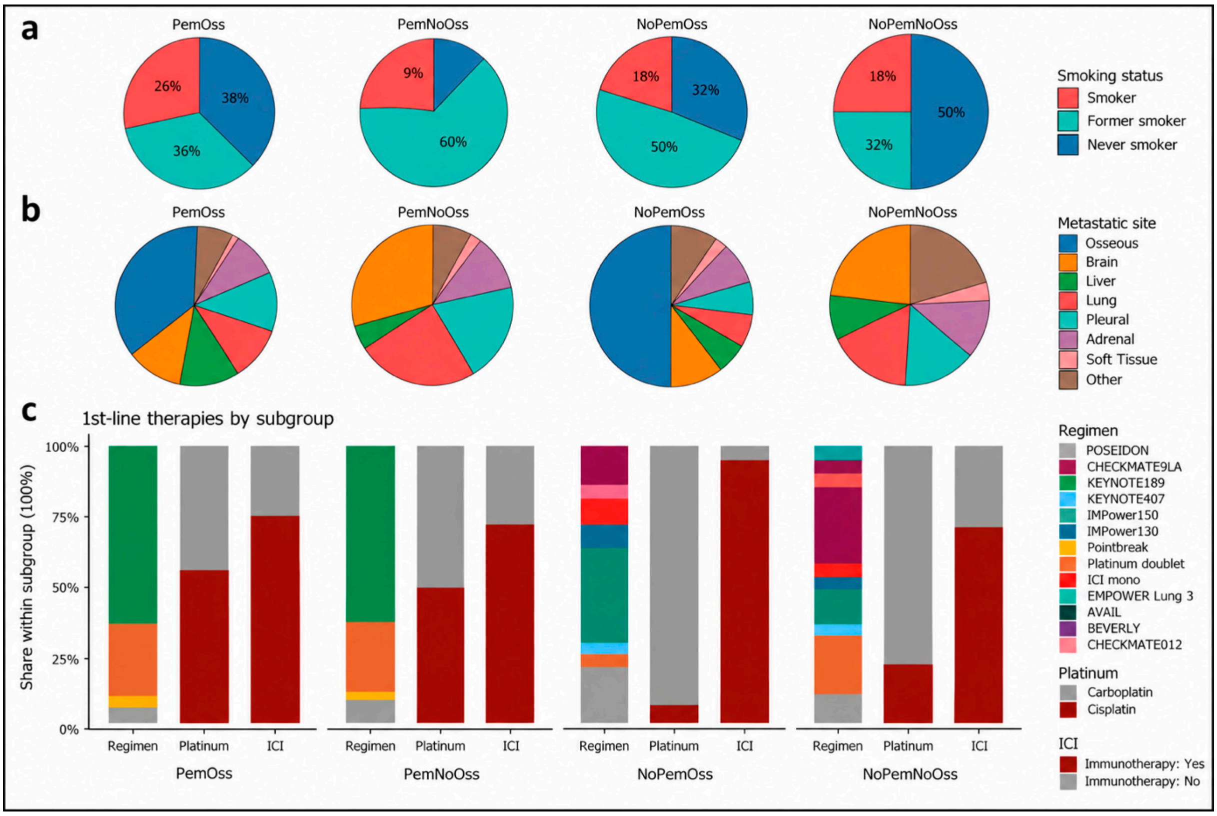

Supplementary Figure S3. Clinical characteristics and treatment distribution across subgroups.

(a) Smoking status distribution in the four study subgroups *(PemOss, PemNoOss, NoPemOss, NoPemNoOss)*. **(b)** Distribution of metastatic sites by subgroup, illustrating distinct metastatic patterns among patients with and without bone involvement. **(c)** Composition of first-line treatment regimens across subgroups. Stacked bars depict the relative frequency of specific chemotherapy–immunotherapy combinations (left), the type of platinum backbone used (middle), and the use of immune checkpoint inhibitors (right). Regimen abbreviations correspond to established clinical trial protocols.

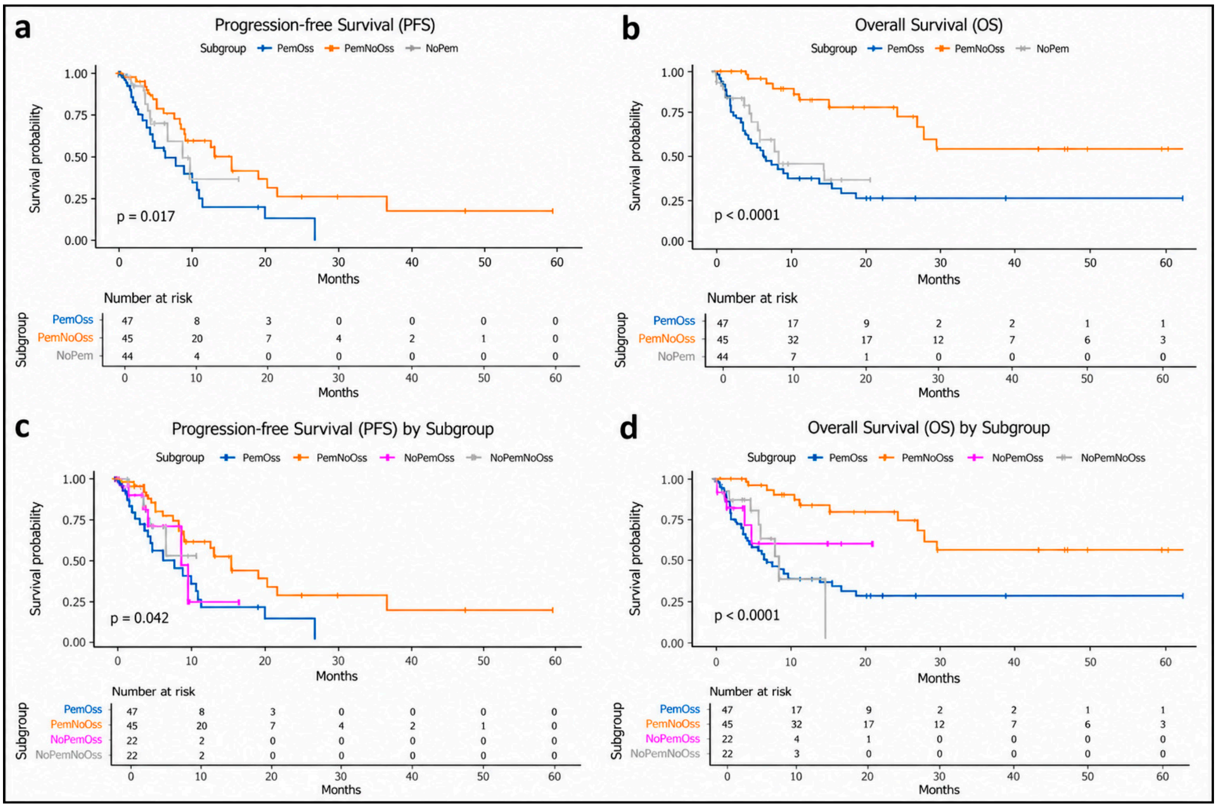

Supplementary Figure S4. Progression-free and overall survival according to pemetrexed treatment and bone metastasis status.

(a) Kaplan–Meier curves for progression-free survival (PFS) comparing patients with bone metastases treated with pemetrexed (PemOss) to those without osseous involvement (PemNoOss) and to non-pemetrexed controls (NoPem). Patients in the PemOs*s* subgroup demonstrated significantly shorter PFS (p = 0.017). **(b)** Overall survival (OS) analysis of the same subgroups revealed a markedly inferior outcome for PemOss patients compared with both PemNoOss and NoPem groups (p < 0.0001). Numbers at risk are displayed below each plot. **c)** Kaplan–Meier curves depicting PFS among the four study subgroups (PemOss, PemNoOss, NoPemOss, NoPemNoOss). Patients treated with pemetrexed and concurrent osseous metastases (*P*emOss) demonstrated significantly shorter PFS compared with all other groups (p = 0.042). **(d)** OS analysis showing the same subgroup stratification. The PemOss cohort exhibited markedly reduced OS compared with the PemNoOss, NoPemOss, and NoPemNoOs*s* subgroups (p < 0.0001). Numbers at risk are indicated below each plot.

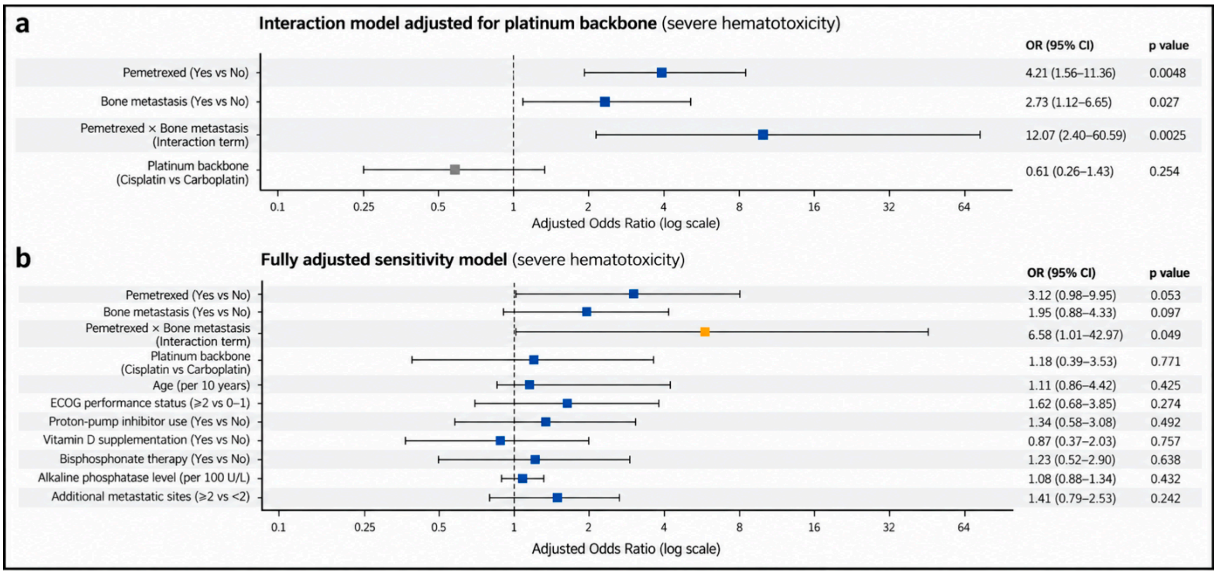

Supplementary Figure S5. Sensitivity analyses accounting for platinum backbone. **(a)** Logistic regression model evaluating the interaction between pemetrexed exposure and bone metastasis status after additional adjustment for platinum backbone (cisplatin vs. carboplatin). **(b)** Fully adjusted sensitivity model additionally including age, ECOG performance status, proton-pump inhibitor use, vitamin D supplementation, bisphosphonate therapy, alkaline phosphatase levels, and additional metastatic sites. Odds ratios (ORs) are presented with 95% confidence intervals. The dashed vertical line indicates OR = 1.

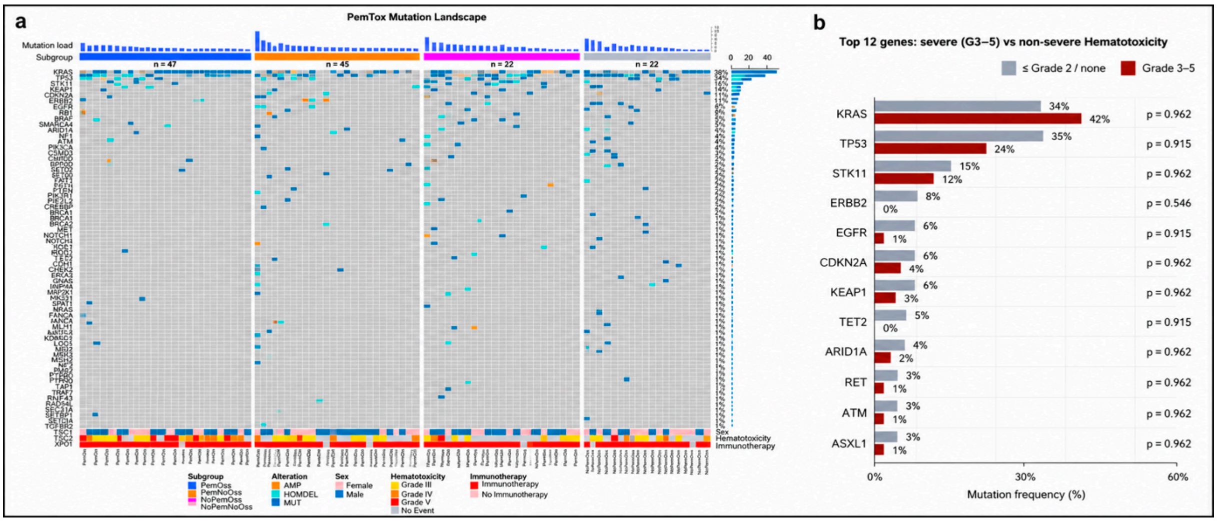

Supplementary Figure S6. Genomic landscape and molecular correlates of severe hematotoxicity.

(a) Oncoprint summarizing the mutational landscape of the cohort stratified by treatment subgroup (*PemOss, PemNoOss, NoPemOss, NoPemNoOss*). Recurrently altered genes comprised ***KRAS, TP53, STK11, CDKN2A***, and ***KEAP1***, reflecting canonical genomic drivers of lung adenocarcinoma. The upper histogram indicates the cumulative mutational burden per case, while the lower annotation bars depict treatment subgroup, presence of bone metastases, and administration of immunotherapy. **(b)** Comparative analysis of the twelve most frequently mutated genes between patients with and without severe hematotoxicity (CTCAE grade III-V). None of the analyzed genomic alterations reached statistical significance, indicating that severe hematotoxicity occurs largely independent of the underlying tumor mutational profile.

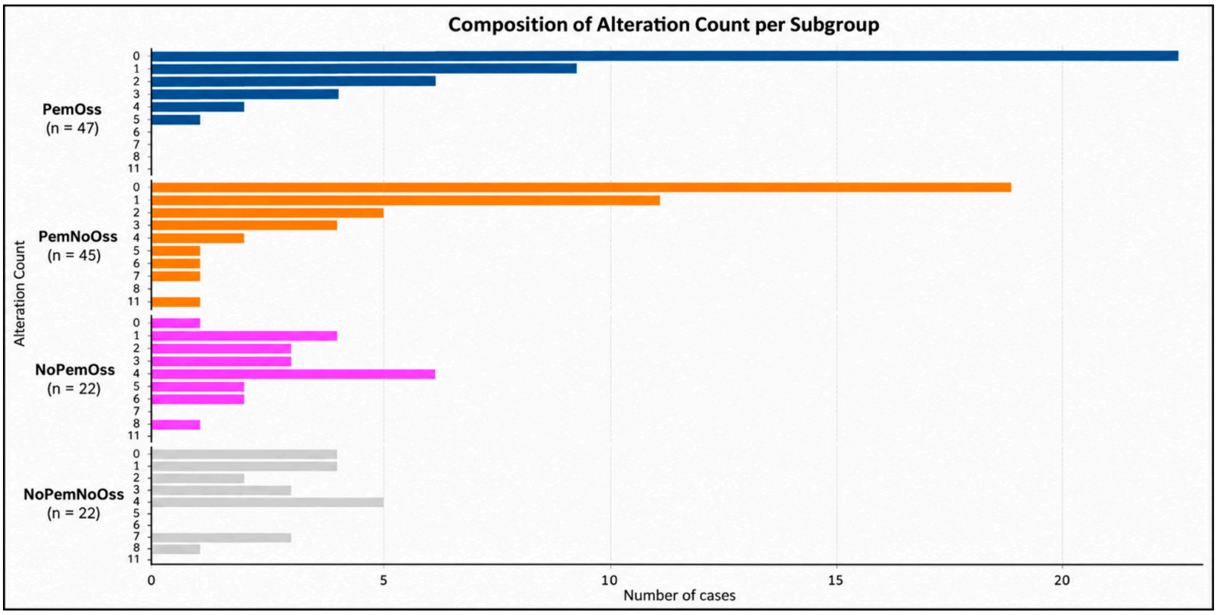

Supplementary Figure S7. Distribution of alteration counts across treatment subgroups. Bar plot illustrating the composition of genetic alteration counts per subgroup (PemOss, PemNoOss, NoPemOss, NoPemNoOss). Each bar represents the number of cases harboring a given count of molecular driver alterations. The PemNoOss subgroup demonstrated the highest overall number of cases with multiple driver alterations, whereas the PemOss and non-pemetrexed subgroups exhibited a broader but less frequent distribution of alteration counts.

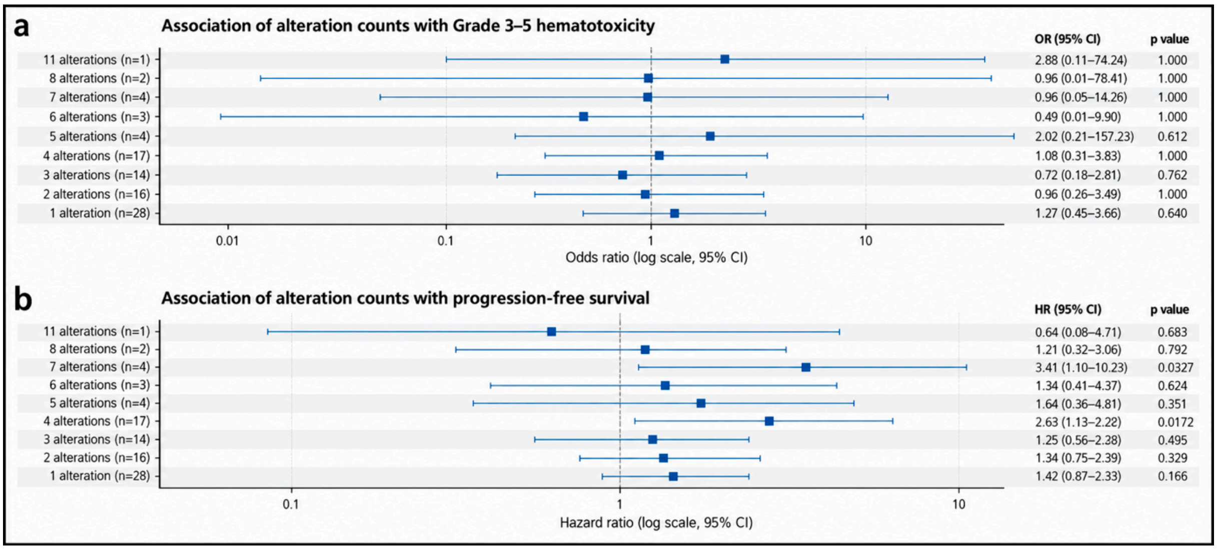

Supplementary Figure S8. Association of potential driver alteration counts with hematotoxicity and progression-free survival.

(a) Forest plot illustrating the association between the number of potential driver alterations per case and the risk of severe hematotoxicity (CTCAE grade III–V). Odds ratios (OR) with 95% confidence intervals are shown on a logarithmic scale. No significant association was observed between alteration count and the incidence of severe hematotoxicity across the analyzed subgroups.

(b) Association between the number of potential driver alterations and PFS, depicted as hazard ratios (HR) with 95% confidence intervals. A statistical trend toward inferior PFS was observed in cases harboring four or more potential driver alterations, suggesting that an increased mutational complexity may be associated with adverse clinical outcomes.

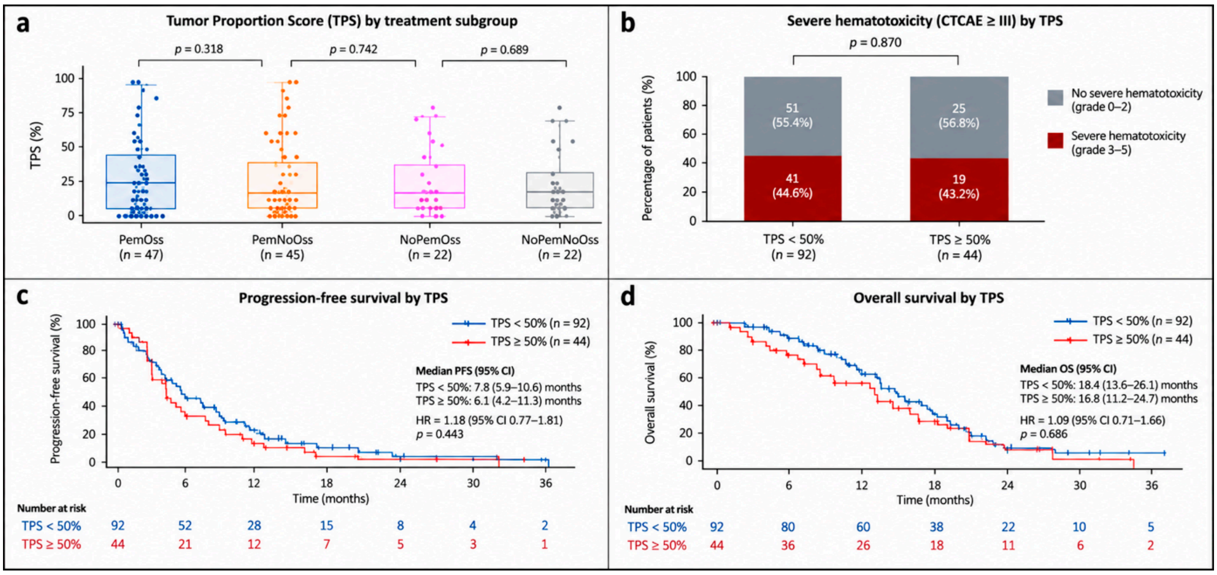

Supplementary Figure S9. PD-L1 tumor proportion score, severe hematotoxicity, and clinical outcomes according to PD-L1 expression. **(a)** Distribution of PD-L1 tumor proportion score (TPS) across the four treatment subgroups: patients receiving pemetrexed with osseous metastases (PemOss), pemetrexed without osseous metastases (PemNoOss), non-pemetrexed treatment with osseous metastases (NoPemOss), and non-pemetrexed treatment without osseous metastases (NoPemNoOss). **(b)** Frequency of severe hematotoxicity (CTCAE grade III–V) according to PD-L1 expression categorized as TPS <50% versus TPS ≥50%. **(c)** Kaplan–Meier analysis of progression-free survival (PFS) according to PD-L1 expression (TPS <50% versus TPS ≥50%). **(d)** Kaplan–Meier analysis of overall survival (OS) according to PD-L1 expression (TPS <50% versus TPS ≥50%). Survival curves were compared using the log-rank test. Hazard ratios (HRs) are presented with 95% confidence intervals (CIs). Numbers at risk are shown below the Kaplan–Meier plots.

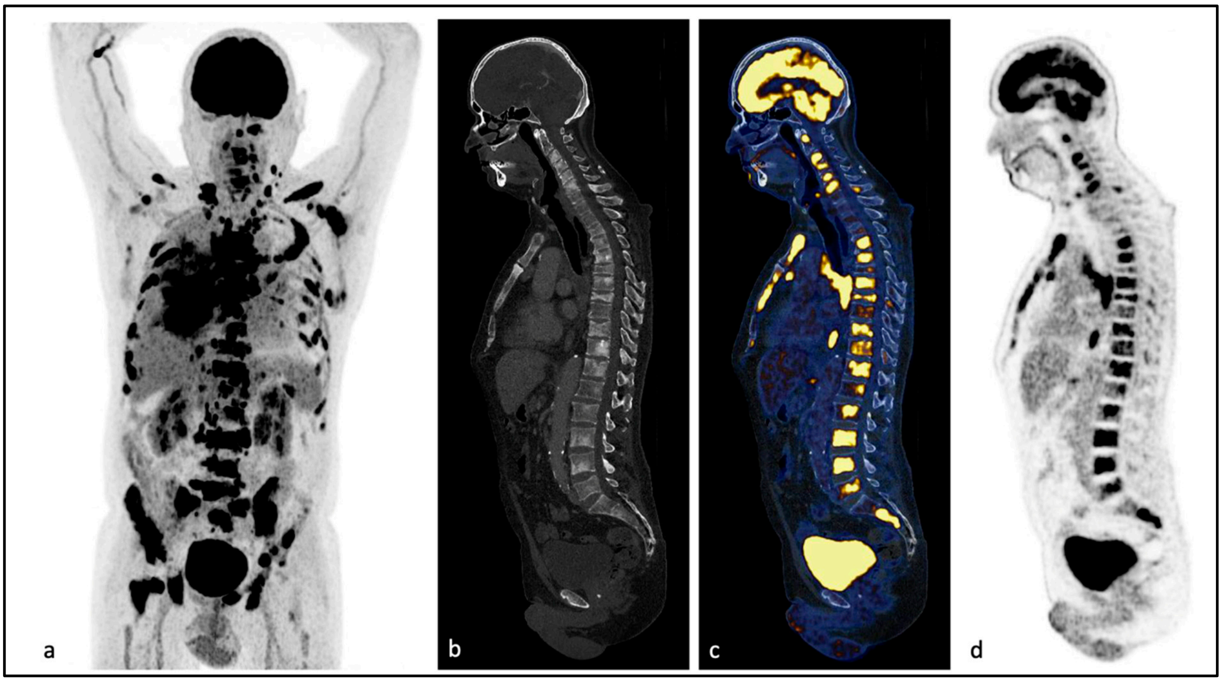

Supplementary Figure S10. PET/CT scan of a patient with diffuse bone metastases and elevated MTV/MTVoss (382/205 cm^3) and TLG/TLGoss (2714/1423 SUVxcm^3). The images show a maximum intensity projection (MIP) of the whole body PET scan (a) and sagittal views in CT (b), PET/CT fusion (c) and in PET (d) revealing diffuse, highly metabolically active bone metastases.

Supplementary References

1. Oken MM, Creech RH, Tormey DC, Horton J, Davis TE, McFadden ET, et al. Toxicity and response criteria of the Eastern Cooperative Oncology Group. Am J Clin Oncol. 1982;5(6):649-55.
2. Rosen RD, Sapra A. TNM Classification. StatPearls. Treasure Island (FL) ineligible companies. Disclosure: Amit Sapra declares no relevant financial relationships with ineligible companies.2025.
3. Tsao MS, Rosenthal A, Nicholson AG, Detterbeck F, Eberhardt WEE, Lievens Y, et al. The International Association for the Study of Lung Cancer Staging Project: The Database and Proposal for the Revision of the Staging of Pulmonary Neuroendocrine Carcinoma in the Forthcoming Ninth Edition of the TNM Classification for Lung Cancer. J Thorac Oncol. 2025;20(7):856-70.
4. Trotti A, Colevas AD, Setser A, Rusch V, Jaques D, Budach V, et al. CTCAE v3.0: development of a comprehensive grading system for the adverse effects of cancer treatment. Semin Radiat Oncol. 2003;13(3):176-81.
5. Nioche C, Orlhac F, Boughdad S, Reuze S, Goya-Outi J, Robert C, et al. LIFEx: A Freeware for Radiomic Feature Calculation in Multimodality Imaging to Accelerate Advances in the Characterization of Tumor Heterogeneity. Cancer Res. 2018;78(16):4786-9.
6. Lopci E, Hicks RJ, Dimitrakopoulou-Strauss A, Dercle L, Iravani A, Seban RD, et al. Joint EANM/SNMMI/ANZSNM practice guidelines/procedure standards on recommended use of [(18)F]FDG PET/CT imaging during immunomodulatory treatments in patients with solid tumors version 1.0. Eur J Nucl Med Mol Imaging. 2022;49(7):2323-41.
7. World Medical A. World Medical Association Declaration of Helsinki: ethical principles for medical research involving human subjects. JAMA. 2013;310(20):2191-4.
